# Dynamics in the Intact fd Bacteriophage Revealed by Pseudo 3D REDOR-Based Magic Angle Spinning NMR

**DOI:** 10.1021/jacsau.4c00549

**Published:** 2024-08-26

**Authors:** Orr Simon Lusky, Dvir Sherer, Amir Goldbourt

**Affiliations:** † School of Chemistry, Faculty of Exact sciences, Tel Aviv University, Tel Aviv 6997801, Israel; ‡ The Center for Physics and Chemistry of Living Systems, Tel Aviv University, Tel Aviv 6997801, Israel

**Keywords:** magic-angle spinning, solid-state NMR, protein dynamics, filamentous bacteriophage, order parameter, dipolar coupling

## Abstract

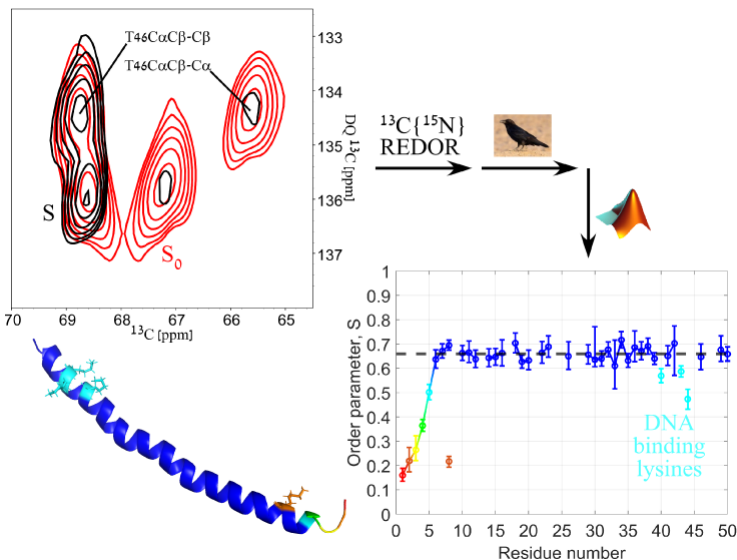

The development of robust NMR methodologies to probe dynamics on the atomic scale is vital to elucidate the close relations between structure, motion, and function in biological systems. Here, we present an automated protocol to measure, using magic-angle spinning NMR, the effective ^13^C–^15^N dipolar coupling constants between multiple spin pairs simultaneously with high accuracy. We use the experimental dipolar coupling constants to quantify the order parameters of multiple C–N bonds in the thousands of identical copies of the coat protein in intact fd-Y21M filamentous bacteriophage virus and describe its overall dynamics on the submillisecond time scale. The method is based on combining three pseudo three-dimensional NMR experiments, where a rotational echo double resonance (REDOR) dephasing block, designed to measure internuclear distances, is combined with three complementary ^13^C–^13^C mixing schemes: dipolar-assisted rotational resonance, through-bond transfer-based double quantum/single quantum correlation, and radio frequency driven recoupling. These mixing schemes result in highly resolved carbon spectra with correlations that are created by different transfer mechanisms. We show that the helical part of the coat protein undergoes a uniform small (∼30°) amplitude motion, while the N-terminus is highly flexible. In addition, our results suggest that the reduced mobility of lysine sidechains at the C-terminus are a signature of binding to the single stranded DNA.

## Introduction

Dynamic fluctuations of biomolecules are fundamental to their function. Examples include enzymatic catalysis and self-assembly processes that necessitate mobility of essential constituents. Elucidating such dynamic behaviors is therefore of great benefit for understanding many biological processes, an example of which is the life cycle of viruses. A virus has to attach to the recognition site in the host cell, inject its genetic material (either DNA or RNA) and the genome must be replicated and translated to generate new viral proteins. Once the proteins are produced, they undergo self-assembly with the genetic material to create new virions capable of infecting new cells. All of these stages necessitate different degrees of molecular flexibility, with variations in the time scales and the amplitudes of motion. Characterizing these motions offers invaluable information on the processes that govern viral replication.

Bacteriophages (phages) are viruses that infect bacteria. Filamentous bacteriophages of family *inoviridae* contain a circular ssDNA genome encapsulated by thousands of identical copies of gVIIIp, the major coat protein. The Ff filamentous phages (fd, f1, M13) infect *E. coli* bearing F-pili.^1^ Their capsid, which mainly contains gVIIIp, is arranged from pentamers related by a 2-fold screw axis (class-I symmetry) that wrap the ssDNA. Ff phages were thoroughly studied as a model system for understanding phage structure,^2−4^ for studying viral morphogenesis^5,6^ and for soft matter physics.^7,8^ Filamentous phages have also proven useful for various applications such as phage display^9,10^ and electrochemistry.^11,12^ Considering the ubiquitous possible usages of these viruses, it is beneficial to establish robust methods to study the property of dynamics in such systems.

Magic angle spinning solid-state NMR (ssNMR) has proven to be a powerful tool for studying dynamics in biomolecules. The ability to probe motions at different time scales was extensively reviewed^13−15^ and was demonstrated on various systems such as amyloids,^16,17^ membrane proteins,^18,19^ and viral proteins assemblies.^20−22^ One of the methods to quantify dynamics in molecules is by measuring motionally averaged anisotropic interactions. The order parameter, *S*, describes the ratio between the breadth of the experimental interaction tensor to its equivalent in the rigid case, when the motion is sufficiently faster than the time scale of the interaction under investigation. Naturally, the values of *S* vary between 1 (rigid limit) and 0 (isotropic motion). When assuming a cylindrical-symmetric motion (“diffusion on a cone”),^23^ the order parameter can be related to the amplitude of motion, θ, according to
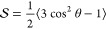
1

In the “diffusion in a cone” model,^24^ the order parameter is related to the maximal angle θ by
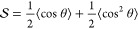
2

Other types of motions can also be modeled and simulated.^25−27^

The calculation of  is straightforward when measuring the motionally averaged dipolar coupling tensor, as the rigid limit is well-defined by the direct dependence of the dipolar coupling constant *d* on the inverse cube of the distance *r*, d = γ_I_γ_S_μ_0_/(4πr^3^) (γ_*I*_ and γ_*S*_ are the gyromagnetic ratios of spins *I* and *S* respectively, and other symbols have their usual meanings). Examples for studying dynamics using averaged dipolar coupling tensors span different types of proteins and their complexes.^28−32^ Early studies utilized mainly the dipolar chemical shift correlation (DIPSHIFT) technique,^33,34^ and other studies utilized various symmetry-based sequences to recouple dipolar couplings.^35−38^ Recently it was shown that DIPSHIFT, and the robust and accurate rotational-echo double resonance (REDOR) technique to measure effective heteronuclear dipolar coupling constants,^39^ are in fact theoretically similar.^40^ In order to implement REDOR two experiments are required – a reference echo experiment (with a signal denoted as “*S*_0_”) and another experiment in which dephasing pulses are employed on a coupled spin (with a signal denoted as “*S*”). The dephasing depends on the dipolar coupling constant, and for a system of two half-spins the resulting REDOR recoupling curve (for ideal pulses) follows the analytical expression^41^
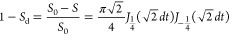
3

Here “*d*” is the effective dipolar coupling constant and “*t*” is the dephasing time. By performing multiple experiments at different dephasing times, the signal can be fitted to eq 3. For small molecules with a well-resolved 1D spectrum, a set of several 1D experiments spanning the initial rise up to 1 – *S*_d_ ≈ 0.6 is sufficient for obtaining accurate dipolar coupling constants. However, for proteins with hundreds of spins, the addition of more dimensions greatly enhances the spectral resolution. Pseudo three-dimensional (p3D) experiments with various mixing schemes, in which the pseudo-dimension is REDOR, were proven to be useful in calculating distances and probing dynamics in specific sites in biomolecules: ^13^C{^19^F} REDOR with radio frequency drive recoupling (RFDR) mixing,^42^^13^C{^2^H} REDOR with CORD mixing^43^ and 2D heteronuclear-resolved ^13^C{^1^H}, ^15^N{^1^H} REDOR^32^ experiments – all demonstrated on the model protein GB1. The latter was used for measuring the order parameters of most backbone N–H and Cα–H bonds in the fully protonated protein using ^1^H detection.

Generally, in order to observe a reduction in the dipolar interaction, the motion must have a sufficiently large (≳10°) amplitude and be at least an order of magnitude faster than the typical time scale of that interaction. As a result, in comparison to measurements of ^1^H–^15^N and ^1^H–^13^C order parameters, the range of motions that can be detected by measuring the effective ^13^C–^15^N dipolar coupling constants is one order of magnitude slower than those involving protons, namely–longer correlation times. Kashefi et al. applied ^13^C{^15^N} REDOR with DARR mixing to study the average couplings of Cα–N and C–N in the peptide MLF and in the cytoplasmic fragment of the *E. coli* aspartate receptor (CF).^44^ They showed that the REDOR block can be used as a filter for a DARR experiment, providing information on the ratio of rigid and dynamic residues. Peak volume analysis of different crosspeaks in this REDOR-DARR experiment reports on this ratio and such an analysis was used to quantify the distribution of mobile residues in CF. Yet, site-specific order parameters have not been derived.

Similar experiments involving a combination of REDOR with various different homonuclear mixing sequences can be used to quantify to high accuracy the individual order parameters of each residue in the protein if the experiments are recorded in a p3D fashion and the buildup curves are site-specifically analyzed. The choice of mixing scheme is important for the type of observed correlations. Mixing based on dipolar interaction can show more correlations, as no chemical bond is required, and since the dipolar interaction is generally stronger than the scalar coupling. However, large-amplitude motions average the dipolar interaction, causing the mixing to be less efficient, and with the effects of dipolar truncation, dynamic spins may not appear at sufficient sensitivity after the mixing period. Scalar-based experiments on the other hand are less compromised by dynamic spins due to their longer transverse relaxation times and due to the fact that this interaction is not averaged out by motion. Thus, such experiments can significantly enhance correlations that do not appear in dipolar-based experiments. Experiments with isotropic mixing like RFDR^45^ utilize both the dipolar and the scalar interactions as transfer mechanisms, allowing observation of both rigid and dynamic regions. For example, with RFDR and short mixing times we could clearly observe crosspeaks of residues 1–3 of the fd coat protein.^46^ The INADEQUATE experiment,^47^ adapted to ssNMR,^48^ is another possible candidate for such studies. The double-quantum (DQ) frequencies observed in the indirect dimension reduce the spectral congestion and facilitate the assignment process. This property, along with its enhanced sensitivity to detect dynamic regions when DQ excitation is based on scalar-coupling, was also shown to aid the assignment of mobile residues in fd coat protein.^49^ Therefore, a combination of REDOR as a dephasing block with INADEQUATE mixing is an adequate candidate for quantitatively studying highly dynamic residues in viruses and other biological macromolecules and complexes. The three mixing schemes are therefore complementary in terms of their information content.

In this study we present the dynamics profile of C–N bonds in the major coat protein of the fd phage possessing a Y21M mutation in gVIIIp. We combined DARR, RFDR, and INADEQUATE mixing schemes with a REDOR dephasing block in order to quantify the amplitudes of motion of Cα-N bonds in the backbone of 36 of its 50 residues (including the highly flexible N-terminus) and of several sidechains containing C–N moieties. We demonstrate that overall, the helix has uniform order parameters, and the N-terminus undergoes large-amplitude motions with an order parameter as low as 0.16. Analysis of sidechain dynamics revealed that the single tryptophan is highly rigid, and that the lysine residues next to the ssDNA exhibit a somewhat restricted motion compared to other sidechains like K8 and Q15, although this motion is of larger amplitudes than the backbone. Finally, we demonstrate the utility of REDOR as a filter to simplify INADEQUATE spectra.

## Materials and Methods

### Sample

A uniformly ^13^C, ^15^N labeled sample of fd-Y21M bacteriophage was prepared using our lab protocols,^50^ and packed into a 4 mm ZrO_2_ MAS rotor.

### NMR Methods

Magic-angle spinning NMR experiments were conducted on a Bruker Avance III spectrometer operating at 14.1T, equipped with a MAS 4 mm E-free probe. The chemical shifts of ^13^C were externally referenced to adamantane at 40.48 ppm.^51^ Experiments were conducted at an estimated temperature of 11 ± 1 °C, calibrated according to the chemical shift of water protons in the sample. The complete list of all experimental parameters appears in Section S1.

### Data Analysis

NMR data were processed using NMRPipe.^52^ The analysis of spectra was done using SPARKY 3.134^53^ and POKY.^54^ RAVEN^55^ was used for crosspeak analysis and for generating buildup curves, which were fit to the universal REDOR curves using a home-written MATLAB script (see Section S2). Linear prediction was used in the indirect dimension of the REDOR-RFDR and INADEQUATE-REDOR experiments. The validity of obtaining quantitative results when using linear prediction is discussed in Section S4.

The identification of spin-pairs belonging to different crosspeaks in REDOR-DARR and REDOR-RFDR experiments relied on our prior assignment of the fd-Y21M phage coat protein^50^ (BMRB entry 26910). The assignment table and the list of the crosspeaks from all the spectra were loaded into the RAVEN software and automatically assigned. The assigned crosspeaks were then analyzed using the “Cross Peaks Analysis Tool” of RAVEN resulting in a table of intensities, containing *S*(*t*) and *S*_0_(*t*) for each crosspeak. Several crosspeaks were analyzed manually (peaks were identified and the intensity was extracted) in order to validate the automated RAVEN process.

Since the current version of RAVEN does not support peak identification from DQ data, as it relies on the BMRB assignment table, we wrote a Python script as an extension to support scalar-coupling-based double-quantum frequencies thus enabling analysis of our INADEQUATE-REDOR data. The script creates a new table from the original BMRB assignment table that includes all possible ^1^J_CC_-based DQ resonances. The revised assignment list can then be used as input for both RAVEN and POKY. The script is available for download via Github, https://github.com/amirgoldbourt/DQ-extension-for-RAVEN-and-POKY. A more detailed description of the script appears in Section S3 and demonstrated in the Supplementary Video POKY and Supplementary Video RAVEN.

In our analysis of the REDOR recoupling curve, we fit the crosspeak intensities from the homonuclear spectra acquired at different mixing times to the universal curve, eq 3, using a home-written MATLAB script (given in Section S2). This analysis inherently assumes a two-spin system and the application of ideal pulses. In order to check the validity of the first assumption, we used the NMR simulation package SIMPSON^56^ to calculate a REDOR recoupling curve for a three-spin system consisting one ^13^C and two ^15^N spins, with dipolar coupling constants of 1005 and 200 Hz, corresponding to the typical distances between Cα and the two N spins of the same and the adjacent amino acids. The fit of the simulated data to the universal curve corresponding to an isolated Cα–N spin pair in the same amino acid resulted in a deviation of 2 Hz, which is smaller than the experimental error, thus validating the approximation. Similarly, simulating a C_3_N spin system containing homonuclear ^13^C–^13^C dipolar couplings of Cα with C and Cβ resulted in a deviation of 4 Hz from the fit to eq 3. The results of the simulations are shown in Section S4. The second assumption ignores the finite pulse correction^57^ (underestimates the dipolar coupling constant by ∼2% for our experimental conditions) when using the universal curve, however, the fit error due to the experimental noise averages ∼5–10% (as derived from χ^2^ fitting) and is higher. This error mainly includes the effect of imperfect π pulses and instability in the spinning speed.

Once all dipolar coupling constants were obtained, the order parameter was calculated as
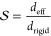
4with *d*_rigid_ being the dipolar coupling constant at the rigid limit, 1005 Hz, determined by the bond length of Cα–N (1.45 Å).^58^ An example of the data analysis appears in the Section S5.

## Results and Discussion

**Figure 1:**
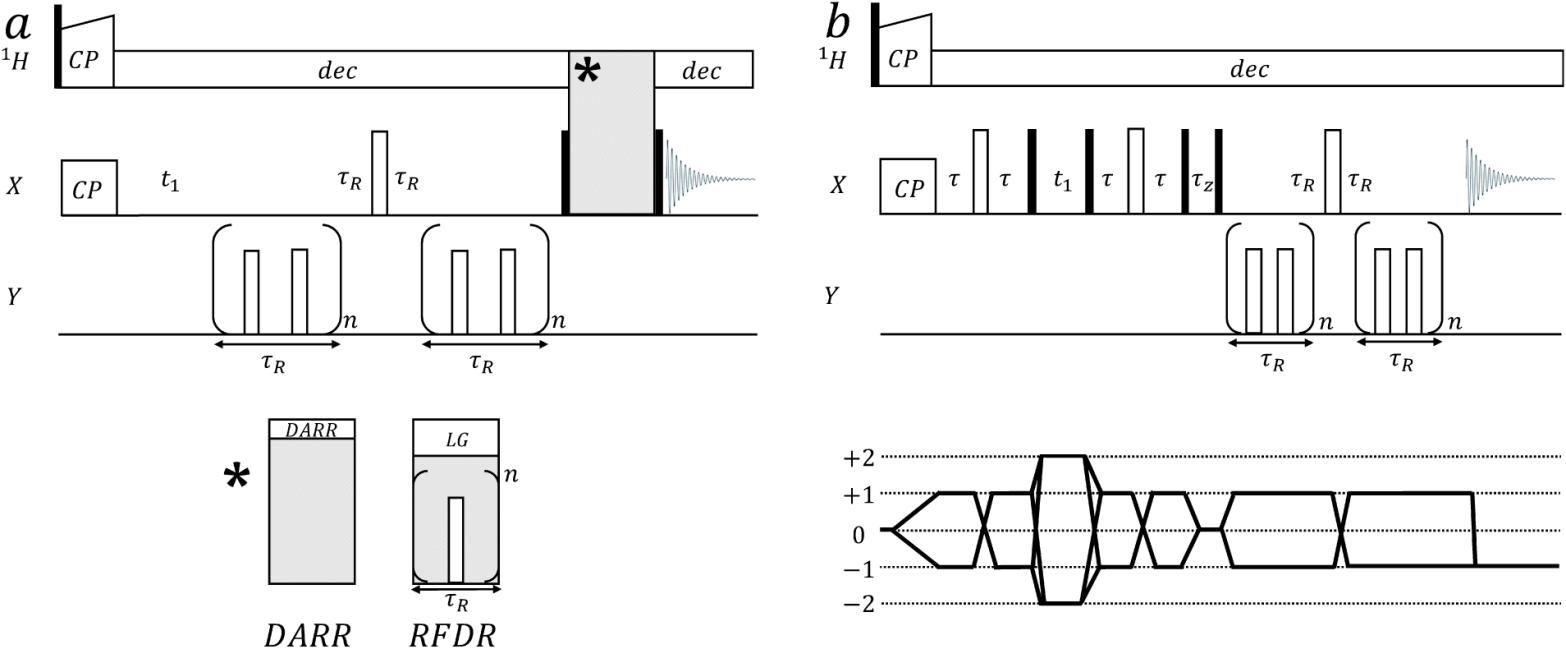
Pseudo 3D pulse sequences for the measurement of 2D-resolved N–C dipolar couplings. Black and white rectangles represent 90° and 180° pulses, respectively. CP stands for cross-polarization block, “dec” stands for ^1^H decoupling and *τ*_R_ is the MAS rotor period. (a) X{Y}–X REDOR-DARR and X{Y}–X REDOR-RFDR pulse sequences. The two applied schemes are identical with the exception of the mixing block represented by the gray rectangle. For DARR, a ^1^H radio frequency irradiation with a strength of *v*_1_ is applied during mixing at the rotary resonance condition . For RFDR, rotor-synchronous 180° pulses are applied on the X channel (^13^C in our experiments) and a homonuclear ^1^H Lee–Goldburg decoupling is employed. (b) X–X{Y} INADEQUATE-REDOR sequence. The delay time τ was optimized for maximal transfer, *τ*_z_ is the z-filter duration to eliminate nonlongitudinal terms. The coherence transfer pathway diagram is drawn beneath. The full phase cycling can be found in Figure S1.

**Figure 2:**
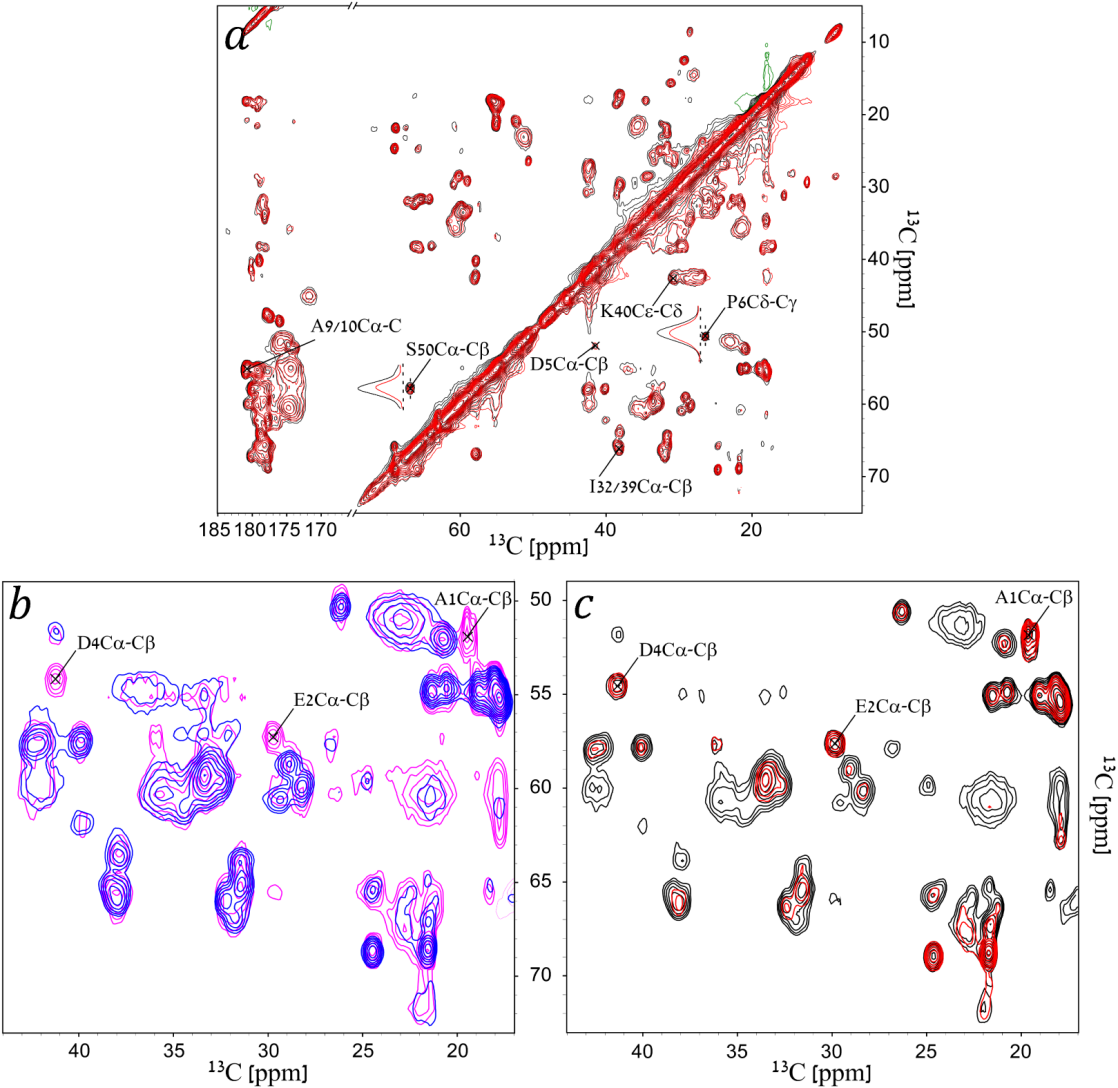
(a) An overlay of 2D REDOR-DARR spectra of gVIIIp in fd-Y21M phage – “*S*_0_” in black and “*S*” in red. The REDOR dephasing time here is 571 μs. Contour levels are drawn at multiples of 1.4 with the lowest matching an SNR of 6. Green represents negative signals. The labels show the assignment for a few crosspeaks that were mentioned in this article. 1D traces of two crosspeaks (P6Cδ−Cγ, and S50Cα–Cβ) are drawn to demonstrate the dephasing on the indirect dimension. (b) An overlay of 2D REDOR-DARR (magenta) and REDOR-RFDR (blue) with a REDOR dephasing time of 429 μs demonstrating the emergence of dynamic residues in RFDR. (c) An overlay of 2D REDOR-RFDR “*S*_0_” (black) and “*S*” (red) spectra with a REDOR dephasing time of 1143 μs. In (b) and (c) contour levels are drawn at multiples of 1.4 with the lowest matching an SNR of 10. The labels show the assignment for the N-terminus residues A1, E2 and D4 that were not detected with DARR mixing.

The general scheme of the experiments, shown in Figure 1, includes a 2D ^13^C–^13^C homonuclear mixing sequence along with a REDOR dephasing block. Three mixing schemes were used: DARR, RFDR and INADEQUATE. The DARR and RFDR mixing times were chosen to be 5 and 6 ms respectively to limit the magnetization transfers to spins close in space (≲3 Å), decreasing the total number of crosspeaks and thus reducing ambiguity and increasing crosspeak sensitivity. An alternative approach, when the signal-to-noise ratio is large, is to use long mixing times, thereby generating multiple redundant crosspeaks bearing the same information. In contrast to the DARR and RFDR-based experiments, where the dephasing was in the indirect dimension, the dephasing in the INADEQUATE-based experiment was in the direct dimension to prevent a more complex spin behavior resulting from dipolar dephasing of double-quantum states. Such an experiment was shown suitable for the measurement of torsion angles in rigid systems.^59^ When combining crosspeak assignments from all three experiments, we were able to resolve and analyze 36 (of the total of 50) residues representing all regions of the coat protein. The remaining 14 residues were not assigned due to spectral overlap in the mostly helical coat protein. As expected, crosspeaks of the residues belonging to the nonstructured, dynamic N-terminus either did not appear in the DARR-based experiment at the short mixing time of 5 ms, or had a very low SNR compared to the RFDR- and INADEQUATE-based experiments (Figure 2). On the other hand, peaks belonging to the backbone of residues in the helical part (residues 6 onward) had an excellent SNR in the DARR spectra.

Figure 2 shows an overlay of two typical 2D DARR-resolved, and two 2D RFDR-resolved spectra comparing “*S*_0_” and “*S*”. It demonstrates reduction in signal intensities (red spectrum) for crosspeaks originating from Cα (bottom part of the spectrum) while signal intensities of sidechains are retained since they are not sufficiently close to the ^15^N amide to produce dephasing after 571 μs (8τ_r_). REDOR curves were then generated from these crosspeaks as detailed in Section S5. A summary of all buildup curves is given in Section S6.

### The Overall C–N Bond Dynamics in fd-Y21M Coat Protein

**Figure 3:**
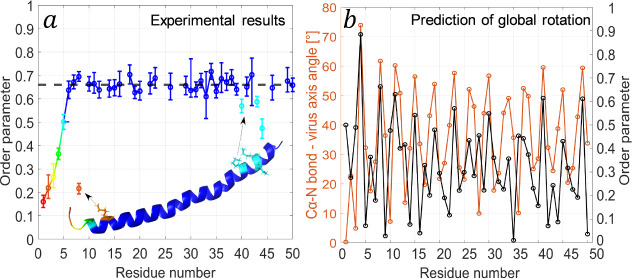
(a) A plot of the order parameters, , of the Cα–N and Cε–N bonds of all residues that appear in at least one of the three types of the p3D REDOR-dephased experiments. The color gradient (red to blue) represents an increasing order parameter. A cartoon of the known structure^2^ (PDB entry: 2C0X) shows a map of the order parameters with the same color scheme. In addition, four lysine residues (8, 40, 43, 44) are shown as sticks to represent the order parameter of their Cε-N bond. The black dashed line represents the average order parameter (0.66 ± 0.02) for residues 6–50, and solid lines represent consecutive residues. (b) A plot of the calculated angles (orange) between the viral filament axis and the Cα–N bond vectors for all residues of one coat protein, derived from the known structure^2^ (PDB entry: 2C0X). In black, predicted order parameters matching the Cα–N angles, calculated according to a motion on a cone model (eq 1). Since the experimental method is insensitive to the sign of the interaction, the absolute value of the order parameters was plotted. The results show that rotational motion around the viral axis on a subms time scale is not consistent with our data.

A total of 142 nonambiguous and 6 ambiguous (with ambiguity of 2) crosspeaks that involve ^13^C spins dephased by ^15^N, from all p3D spectra, were assigned and fit to the REDOR universal curve. Most fits show good agreement to eq 3 with values of . These values show that the assumptions we made, a two-spin system subjected to motions that are faster than the size of the dipolar interaction strength, are acceptable. For the ambiguous crosspeaks (A9–A10, D12–L14, and I32–I39), the fit to a single dephasing curve suggests that the motion of the Cα–N bonds of both amino acids has a similar order parameter. This was also verified by the results of the fit of their corresponding resolved crosspeaks (see Section S6), and is also supported by our previous observations in which the CSA of the backbone spins shared a similar breadth.^55,60^ The experimental order parameters of the backbone Cα–N bonds are plotted in Figure 3a. This plot shows a trend in which the first residues belonging to the N-terminus (residues 1–5) have larger amplitudes of motion, with an increasing order parameter until reaching a plateau. These results are in agreement with previous reports that showed a flexible nonstructured N-terminus.^61,62^ The majority of the protein (residues 6–50) is in a well-defined helix. The average order parameter of the Cα–N bond in the helix is 0.66 ± 0.02 and is lower than order parameters obtained from C–H and even N–H solid-state NMR measurements of other systems such as Pf1 phage coat protein (C–H 0.99 ± 0.04),^63^ GB1 (C–H 0.94 ± 0.04, N–H 0.90 ± 0.07)^64^ and ubiquitin (C–H 0.78 ± 0.09).^30^ When motionally averaged CSA values were measured,^55,60^ we obtained for the helical part an averaged value of ∼12.5 kHz for C′ and ∼6.3 kHz for the ^15^N amide, very similar to GB1,^65,66^ which is considered to be highly rigid. The order parameters we measure here for Cα–N bonds imply dynamics on a slower time scale due to a smaller anisotropy of the Cα–N (∼1 kHz) dipolar coupling constant compared to the CSA.

A plausible global motion is a rotation of the entire virus about the axis of the filament, since the virus in its precipitated form is highly hydrated and forms liquid crystals. Such a rotation implies that the Cα–N bonds rotate in a conic motion, which would translate into oscillations in the order parameter. The angles of those bonds relative to the viral axis can be calculated from the known structure,^2^ and in the case of such a global motion, those angles would represent the amplitudes of motion on a cone. However, by calculating the expected order parameters it is clear that the model fails to describe the results. Figure 3b shows the angles between the Cα–N bonds and the vertical axis of the virus, and the absolute value of the calculated order parameters. The calculated periodicity of the order parameters and the large variations in values do not fit the experimental data, and therefore global rotational motion does not occur at a time scale faster than ∼ms.

An alternative option is a uniform motion, namely, a motion with similar amplitudes and overall trajectories that are shared by residues 6–50. In the context of a viral particle, the simplest form of motion that can be suggested is a jittering motion as a result of thermal fluctuations. It is difficult to model such motions and future studies by molecular dynamics, once a plausible model of the ssDNA is obtained, may demonstrate this result.

### Dynamics of the N-Terminus

**Figure 4:**
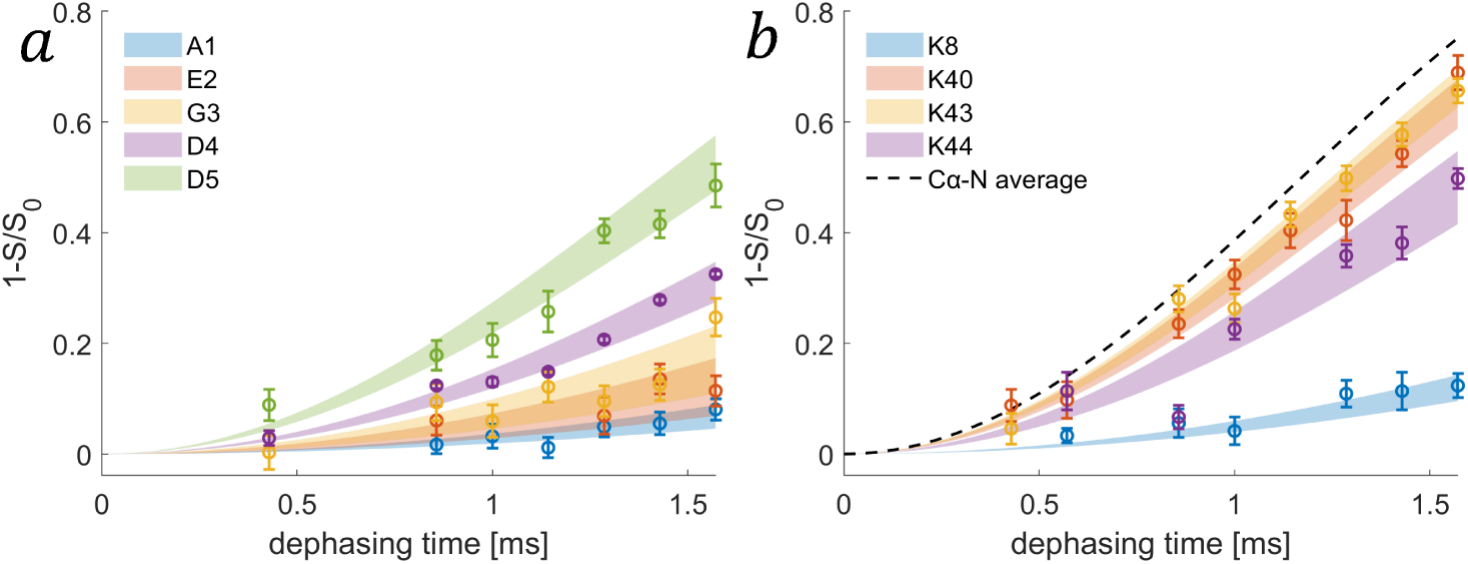
(a) Best-fit^13^Cα{^15^N}-REDOR buildup curves (from all experimental observations) in the N-terminus residues of fd-Y21M coat protein (A1 d= Hz in blue, E2 d= Hz in orange, G3 d= Hz in yellow, D4 d= Hz in purple, and D5 d= Hz in green). (b) REDOR buildup curves for the average effective dipolar coupling constant calculated for lysine residues (K8 d= Hz in blue, K40 d= Hz in orange, K43 d= Hz in yellow, and K44 d= Hz in purple). The dashed curve is the average Cα–N dipolar coupling constant of residues 6–50, 663 Hz. In both (a) and (b) the transparent blocks are the error bars of the fit. The data points are the experimental results, averaged over multiple crosspeaks.

Figure 4a shows the best-fit Cα{^15^N}–CX REDOR buildup curves of residues belonging to the N-terminus. Their values and amplitudes of motion are given in Table 1. Due to the ability of the RFDR experiment to resolve highly dynamic residues, we were even able to observe a quantitative recoupling curve for A1 yielding a very low order parameter of 0.16. As also shown in Figure 3, it is clear that the nonstructured N-terminus exhibits large-amplitude motions compared to the rest of the protein. The monotonic increase in the order parameter, hence in the amplitudes of motion, suggests a gradual change, where the tip exhibits the largest motion. The N-terminus in the structure has a shape of a hook that points outward toward the solvent, and has less inter-residues contacts, possibly allowing the enhanced mobility. REDOR-RFDR spectra showing N-terminus crosspeaks appear in Figure 2.

**Table 1:** Order Parameters of the Cα–N Bonds in the N-Terminus of the fd-Y21M Coat Protein and the Overall Average of Residues 6–50 as a Comparison^a^

residue	order parameter	amplitude (motion on a cone) (deg)	largest amplitude (motion in a cone) (deg)
A1			
E2			
G3			
D4			
D5			
Average			

a The rigid limit was set to be *d*_rigid_ = 1005 Hz, matching a bond length of 1.45 Å. The amplitudes of motion were calculated according to eqs 1 and 2.

### Dynamics of Sidechains

Sidechains of six amino acids contain C–N bonds and therefore their order parameters can also be determined by the proposed p3D methods. They include two positively charged amino acids (R, K), which are commonly involved in binding to negatively charged surfaces such as DNA, or form salt bridges. They also include the polar residues H, Q, and N, and the hydrophobic residue W.

The coat protein of fd-Y21M phage contains five different lysine residues, one tryptophan, and one glutamine. Of the lysines, K8 is located in the start of the helix and is in proximity to the negatively charged D4 and D5, as well as E20 of a neighboring coat protein subunit. Other lysines are located in the C-terminus and are in proximity to the ssDNA.^4,67^ The single tryptophan is located in the hydrophobic packing epitope and interacts with other aromatic residues thus stabilizing the capsid structure.^3^ Q15 is in the core of the helix.

Table 2 summarizes the order parameters of six sidechains (K48 is unresolved). Figure 3a and the REDOR buildup curves shown in Figure 4b, generated by the averaged Cε–Nζ effective dipolar coupling constants of the lysine sidechains, show smaller than average order parameters (0.22 for K8, 0.47–0.59 for DNA binding lysines), in agreement with similar observations in Pf1 phage.^30^ One cause for this enhanced dynamics is that the distance between Nζ and the ssDNA allows for water molecules to bridge between the capsid and the nucleic acid.^68,69^ In fact, we have previously observed that lysine to DNA ribose correlations appear only at long mixing times (500 ms).^70^ Interestingly we observe that K8 is significantly more dynamic than the other lysine sidechains in contact with DNA although it has three negatively charged residues in its vicinity, D4, D5 and E20. Nevertheless, the distances are probably sufficiently long (4.5–5.9 Å) to allow for larger amplitude motions and for the presence of water molecules. These results also suggest that although the lysine–DNA interaction allows for motion despite the electrostatic interactions, it is still more restricted than that of K8, and probably of other sidechains that have motional freedom around the χ_1_, χ_2_, and the other sidechain torsion angles.

**Table 2:** Sidechain Order Parameters in the fd-Y21M Coat Protein: Cε–Nζ Bond in the Lysine Residues, Cδ−Nε_2_ in Q15 and Cε_2_–Nε_1_ in W26^a^

#residue	order parameter
K8	
Q15	
W26	
K40	
K43	
K44	

a The rigid limits for K, Q, and W were set to be *d*_rigid_ = 1005 Hz, 1317 Hz, and 1116 Hz respectively, matching the bond lengths of 1.45 Å, 1.32 Å and 1.40 Å.

The residue W26 was expected to be immobile,^62^ and was even used as a distance reference when solving the structure of the M13 phage.^3^ The order parameter that was obtained from the REDOR curve for the Cε_2_–Nε_1_ bond located in the pyrrole ring has a value of , which is higher than any of the other C–N bonds that were detected in all p3D experiments. This fits the previous description of W26 being significantly more rigid due to its role in stabilizing the hydrophobic core of the viral capsid.^71^

The order parameter of the sidechain Cδ−Nε_2_ bond in Q15 is relatively low, , suggesting large amplitude motions. This fits the structure in which that residue points outward toward the solvent.

### Filtered INADEQUATE

The p3D experiments require the acquisition of multiple REDOR-dephased “*S*” spectra, including such with dephasing times that cause Cα signal loss of 80% and beyond. Since the dephasing is caused by proximity to ^15^N, the majority of the remaining crosspeaks are those of the sidechains. As a result, these spectra have reduced spectral congestion, and can facilitate the process of assignment and the extraction of many other chemical-shift-based properties. For example, it is highly useful for assigning residues containing carbonyl sidechains (D, E, N and Q) that in many cases overlap with the backbone carbonyl signals. D and E residues are unaffected by the REDOR dephasing pulses, and N and Q sidechain carbonyls tend to be more dynamic compared to the backbone, meaning that the dephasing efficiency of their signal is lower. Indeed, REDOR was used in the past as a filter for DQ-SQ dipolar-based spectroscopy to distinguish aspartate and glutamate from aspartic acid and glutamic acid in ubiquitin and in a membrane protein in lipid bilayers.^72^

We show here that scalar-based filtered INADEQUATE is highly useful, since information about Cα is still maintained in the DQ dimension in the form of crosspeaks with Cβ, meaning that it can still be used for assignment. By choosing the dephasing time according to a theoretical dephasing curve corresponding to the Cα–N distance, the rigid atoms can be filtered out of the spectrum facilitating assignment of resonances that may be unresolved in the nonfiltered version.

**Figure 5:**
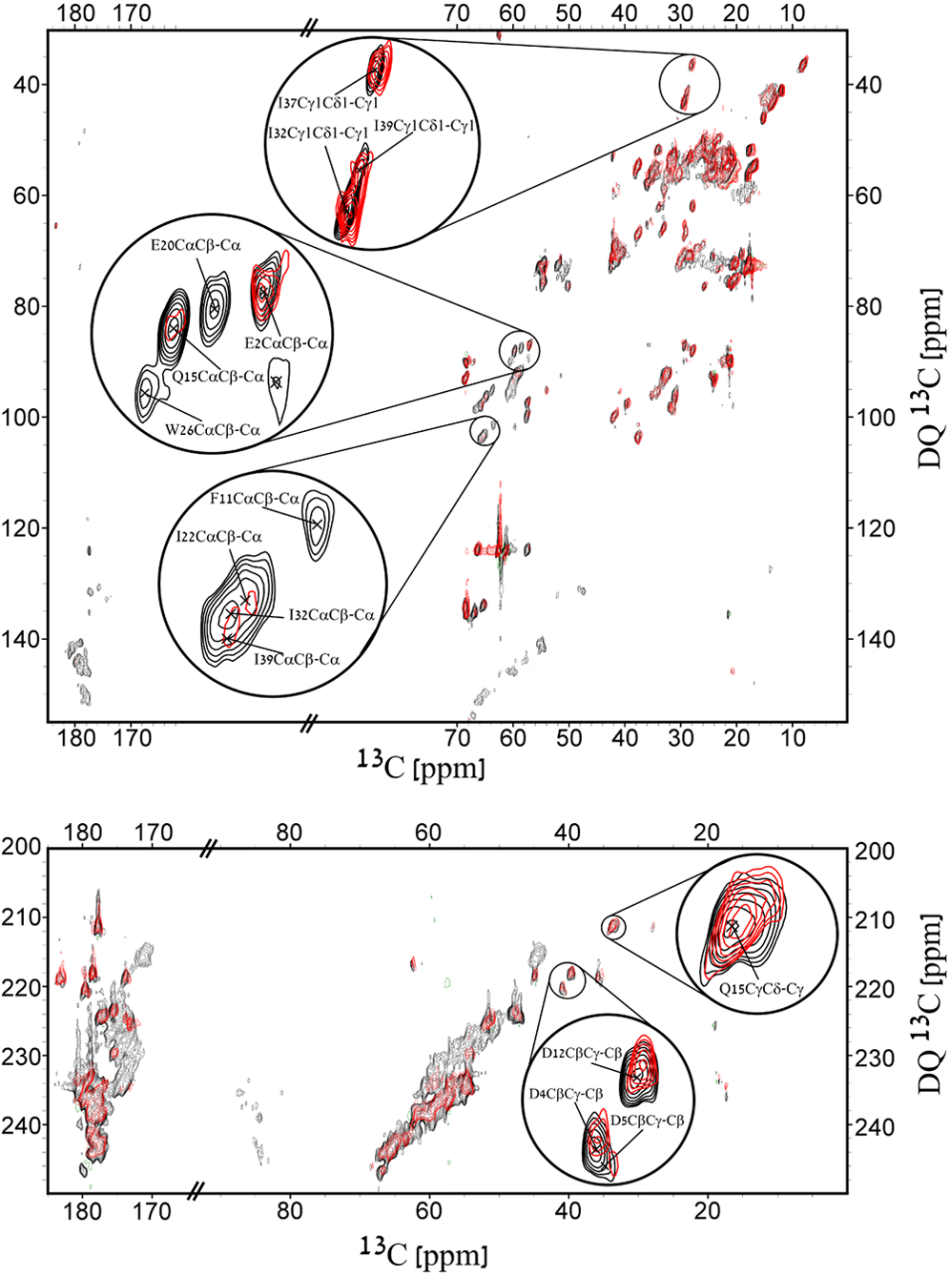
Filtered INADEQUATE-REDOR experiments. The black spectrum is “*S*_0_” with an echo time of 429 μs, and the red is “*S*” with a dephasing time of 1714 μs. The upper and bottom panels show the aliphatic and carbonyl regions of the spectrum respectively, and include magnified crosspeaks in circles. Contour levels are drawn at multiples of 1.3, with the lowest matching an SNR of 6.

Figure 5 shows an overlay of the INADEQUATE-REDOR “*S*” spectrum with the longest dephasing time we recorded, 1714 μs, with that of the “*S*_0_” reference spectrum recorded with a mixing time of 429 μs. The upper panel, showing the aliphatic region, demonstrates the selective dephasing of crosspeaks; The sidechains are retained in the “*S*” spectrum, whereas crosspeaks of Cα undergo dephasing (a site-specific demonstration of threonine residues appears in Section S7). It is also visible that the signal of the crosspeak corresponding to E2CαCβ–Cα only partially dephased due to elevated dynamics compared to the other similar crosspeaks (e.g., the adjacent E20) demonstrating the potential of an INADEQUATE-REDOR filter as a way to differentiate rigid and dynamic regions. The bottom panel shows the carbonyl region, where the REDOR pulses yielded a more resolved spectrum leaving peaks belonging to sidechain amide and carboxylic groups (E2Cδ, D4Cγ, D5Cγ, Q15Cδ), backbone carbonyl of dynamic residues (G3, D4, D5), and some reduced signals of partially dephased crosspeaks with similar shifts (G23, G34, G38). One drawback of the experiment is that the long REDOR echo times inherently cause loss of magnetization due to relaxation. Here an average of 13% decrease in SNR was measured between the reference “*S*_0_” spectrum (429 μs) and “*S*” spectrum (1714 μs) (Section S8). Yet, this phenomena also occurs in other similar REDOR-filtered experiments,^44,73−76^ relaxation-based filters (*T*_1_, *T*_2_, *T*_1ρ_),^77^ etc.

## Summary and Conclusions

In this work we presented a set of three pseudo-3D solid-state NMR experiments to study submilliseconds dynamics in the coat protein of intact fd-Y21M filamentous bacteriophage virus. The dynamics are characterized by the simultaneous measurement and automated analysis of multiple C–N dipolar order parameters, and the experimental strategies are suitable for many similarly large protein complexes. For the fd coat protein, we show enhanced dynamics of the N-terminus, down to an order parameter of 0.16 for the terminal alanine, in excellent agreement with previous measurements of dynamics that were based on recoupling of the chemical shift anisotropy. The majority of the coat protein was assigned (36/50), with the others being unassigned due to spectral overlap as a consequence of the helical character of the protein. We showed reduced dynamics of ssDNA binding lysine residues with respect to other mobile sidechains, probably resulting from water-mediated electrostatic interactions with the negatively charged phosphates. The motion of the indole ring of the tryptophan is shown to be restricted, likely due to strong hydrophobic packing interactions. We showed that a global rotational motion of the entire virus along its axis, at the time scale faster than ms, is not in agreement with our data. The uniform distribution of order parameters along the helix implies that the amplitudes of motion at the sub-ms time scale are shared by all the residues in the helix and we hypothesize that such a motion is a result of some uniform jittering. A more accurate description of the motions that lead to the observed order parameters can potentially be predicted from molecular dynamics, and such studies will be possible once a plausible model for the ssDNA is obtained.

The use of three different mixing schemes – DARR, RFDR, and INADEQUATE, was advantageous in the means of getting access to signals originating from both dynamic and rigid spins since they provide complementary spectra that produce correlations based on both dipolar and scalar couplings. The same protocol can be easily generalized by choosing other mixing schemes tailored to the necessity of the system. In some cases, cross-polarization (CP) may not be efficient for the excitation of dynamic regions even when using long mixing times. In those cases, scalar-based transfer and even direct excitation may be more suitable, however, if the ^1^H–^13^C dipolar coupling is too small to generate polarization via CP, it is highly likely that the effective ^13^C–^15^N dipolar coupling constant is too small to detect with REDOR and the motion is probably close to isotropic. For the fd-Y21M bacteriophage in this study CP was a competent polarization transfer mechanism and all dynamic carbons have been efficiently detected.

Finally, we demonstrated the usefulness of REDOR as a filter for INADEQUATE spectra yielding better resolved spectra in the backbone region. The advantage of the DQ approach here is that information on Cα peaks still exists in CαCβ–Cβ crosspeaks that do not undergo dephasing, unlike SQ-based correlations, where the result is the remaining of one side of the diagonal with a similar density of signals.

While demonstrating our experimental approach on an intact filamentous bacteriophage virus, the method can be easily implemented in the study of other viruses and of additional biomacromolecules such as membrane proteins and amyloids. The ability to probe the dynamics of the positively charged amino acids, all of which include nitrogen atoms in their sidechains, is particularly attractive for studying the properties of biocomplexes where negatively charged protein complexes are common (DNA/RNA, membranes et cetera). In addition, the hurdles of spectral congestion can be overcome for example by using sparsely labeled samples or by resolving the residues of interest by adding additional dimensions. We believe that due to the robustness of this protocol it will be broadly applicable for diverse systems.

## Supporting Information Available

NMR experimental parameters, matlab scripts for fitting the REDOR curves, description of the double-quantum (DQ) extension for RAVEN and POKY software packages, testing of linear prediction validity in quantitative analysis of peak intensities, all dephasing curves and fits to effective dipolar couplings including error analysis, filtered inadequate experimental examples (PDF)
Video showing the use of the DQ extension for POKY (MP4)
Video showing the use of the DQ extension for RAVEN (MP4)

## References

[ref1] Rasched I.; Oberer E. Ff Coliphages: Structural and Functional Relationships. Microbiol Rev. 1986, 50 (4), 401–427. 10.1128/mr.50.4.401-427.1986. 3540571 PMC373080

[ref2] Marvin D. A.; Welsh L. C.; Symmons M. F.; Scott W. R. P.; Straus S. K. Molecular Structure of Fd (F1, M13) Filamentous Bacteriophage Refined with Respect to X-Ray Fibre Diffraction and Solid-State NMR Data Supports Specific Models of Phage Assembly at the Bacterial Membrane. J. Mol. Biol. 2006, 355 (2), 294–309. 10.1016/j.jmb.2005.10.048. 16300790

[ref3] Morag O.; Sgourakis N. G.; Baker D.; Goldbourt A. The NMR–Rosetta Capsid Model of M13 Bacteriophage Reveals a Quadrupled Hydrophobic Packing Epitope. Proc. Natl. Acad. Sci. U. S. A. 2015, 112 (4), 971–976. 10.1073/pnas.1415393112. 25587134 PMC4313819

[ref4] Conners R.; León-Quezada R. I.; McLaren M.; Bennett N. J.; Daum B.; Rakonjac J.; Gold V. A. M. Cryo-Electron Microscopy of the F1 Filamentous Phage Reveals Insights into Viral Infection and Assembly. Nat. Commun. 2023, 14 (1), 2724 10.1038/s41467-023-37915-w. 37169795 PMC10175506

[ref5] Marvin D. Filamentous Phage Structure, Infection and Assembly. Curr. Opin. Struct. Biol. 1998, 8 (2), 150–158. 10.1016/S0959-440X(98)80032-8. 9631287

[ref6] Olszak T.; Latka A.; Roszniowski B.; Valvano M. A.; Drulis-Kawa Z. Phage Life Cycles Behind Bacterial Biodiversity. Curr. Med. Chem. 2017, 24 (36), 3987–4001. 10.2174/0929867324666170413100136. 28412903

[ref7] Tomar S.; Green M. M.; Day L. A. DNA–Protein Interactions as the Source of Large-Length-Scale Chirality Evident in the Liquid Crystal Behavior of Filamentous Bacteriophages. J. Am. Chem. Soc. 2007, 129 (11), 3367–3375. 10.1021/ja068498d. 17316002

[ref8] Dogic Z. Filamentous Phages As a Model System in Soft Matter Physics. Front. Microbiol. 2016, 7, 1013 10.3389/fmicb.2016.01013. 27446051 PMC4927585

[ref9] Smith G. P.; Petrenko V. A. Phage Display. Chem. Rev. 1997, 97 (2), 391–410. 10.1021/cr960065d. 11848876

[ref10] Jaroszewicz W.; Morcinek-Orłowska J.; Pierzynowska K.; Gaffke L.; Węgrzyn G. Phage Display and Other Peptide Display Technologies. FEMS Microbiol. Rev. 2022, 46 (2), fuab052 10.1093/femsre/fuab052. 34673942

[ref11] Oh D.; Qi J.; Lu Y.-C.; Zhang Y.; Shao-Horn Y.; Belcher A. M. Biologically Enhanced Cathode Design for Improved Capacity and Cycle Life for Lithium-Oxygen Batteries. Nat. Commun. 2013, 4 (1), 2756 10.1038/ncomms3756. 24220635 PMC3930201

[ref12] Blaik R. A.; Lan E.; Huang Y.; Dunn B. Gold-Coated M13 Bacteriophage as a Template for Glucose Oxidase Biofuel Cells with Direct Electron Transfer. ACS Nano 2016, 10 (1), 324–332. 10.1021/acsnano.5b04580. 26593851

[ref13] Torchia D. A. Solid State NMR Studies of Protein Internal Dynamics. Annu. Rev. Biophys. Bioeng. 1984, 13 (1), 125–144. 10.1146/annurev.bb.13.060184.001013. 6378066

[ref14] Lewandowski J. R. Advances in Solid-State Relaxation Methodology for Probing Site-Specific Protein Dynamics. Acc. Chem. Res. 2013, 46 (9), 2018–2027. 10.1021/ar300334g. 23621579

[ref15] Schanda P.; Ernst M. Studying Dynamics by Magic-Angle Spinning Solid-State NMR Spectroscopy: Principles and Applications to Biomolecules. Prog. Nucl. Magn. Reson. Spectrosc. 2016, 96, 1–46. 10.1016/j.pnmrs.2016.02.001. 27110043 PMC4836562

[ref16] Scheidt H. A.; Morgado I.; Rothemund S.; Huster D. Dynamics of Amyloid β Fibrils Revealed by Solid-State NMR. J. Biol. Chem. 2012, 287 (3), 2017–2021. 10.1074/jbc.M111.308619. 22130659 PMC3265881

[ref17] Rezaei-Ghaleh N.; Parigi G.; Zweckstetter M. Reorientational Dynamics of Amyloid-β from NMR Spin Relaxation and Molecular Simulation. J. Phys. Chem. Lett. 2019, 10 (12), 3369–3375. 10.1021/acs.jpclett.9b01050. 31181936 PMC6598774

[ref18] McDermott A. Structure and Dynamics of Membrane Proteins by Magic Angle Spinning Solid-State NMR. Annu. Rev. Biophys. 2009, 38 (1), 385–403. 10.1146/annurev.biophys.050708.133719. 19245337

[ref19] Mandala V. S.; Williams J. K.; Hong M. Structure and Dynamics of Membrane Proteins from Solid-State NMR. Annu. Rev. Biophys. 2018, 47 (1), 201–222. 10.1146/annurev-biophys-070816-033712. 29498890 PMC6312106

[ref20] Chen B.; Tycko R. Structural and Dynamical Characterization of Tubular HIV-1 Capsid Protein Assemblies by Solid State Nuclear Magnetic Resonance and Electron Microscopy. Protein Sci. 2010, 19 (4), 716–730. 10.1002/pro.348. 20095046 PMC2867012

[ref21] Lecoq L.; Fogeron M.-L.; Meier B. H.; Nassal M.; Böckmann A. Solid-State NMR for Studying the Structure and Dynamics of Viral Assemblies. Viruses 2020, 12 (10), 1069 10.3390/v12101069. 32987909 PMC7599928

[ref22] Malär A. A.; Callon M.; Smith A. A.; Wang S.; Lecoq L.; Pérez-Segura C.; Hadden-Perilla J. A.; Böckmann A.; Meier B. H. Experimental Characterization of the Hepatitis B Virus Capsid Dynamics by Solid-State NMR. Front. Mol. Biosci. 2022, 8, 807577 10.3389/fmolb.2021.807577. 35047563 PMC8762115

[ref23] Lipari G.; Szabo A. Model-Free Approach to the Interpretation of Nuclear Magnetic Resonance Relaxation in Macromolecules. 1. Theory and Range of Validity. J. Am. Chem. Soc. 1982, 104 (17), 4546–4559. 10.1021/ja00381a009.

[ref24] Lipari G.; Szabo A. Padé Approximants to Correlation Functions for Restricted Rotational Diffusion. J. Chem. Phys. 1981, 75 (6), 2971–2976. 10.1063/1.442388.

[ref25] Wittebort R. J.; Szabo A. Theory of NMR Relaxation in Macromolecules: Restricted Diffusion and Jump Models for Multiple Internal Rotations in Amino Acid Side Chains. J. Chem. Phys. 1978, 69 (4), 1722–1736. 10.1063/1.436748.

[ref26] Torchia D. A.; Szabo A. Spin-Lattice Relaxation in Solids. J. Magn. Reson. 1969 1982, 49 (1), 107–121. 10.1016/0022-2364(82)90301-8.

[ref27] Zumpfe K.; Smith A. A. Model-Free or Not?. Front. Mol. Biosci. 2021, 8, 727553 10.3389/fmolb.2021.727553. 34760924 PMC8573340

[ref28] Barré P.; Zschörnig O.; Arnold K.; Huster D. Structural and Dynamical Changes of the Bindin B18 Peptide upon Binding to Lipid Membranes. A Solid-State NMR Study. Biochemistry 2003, 42 (27), 8377–8386. 10.1021/bi034239e. 12846587

[ref29] Reichert D.; Pascui O.; deAzevedo E. R.; Bonagamba T. J.; Arnold K.; Huster D. A Solid-State NMR Study of the Fast and Slow Dynamics of Collagen Fibrils at Varying Hydration Levels. Magn. Reson. Chem. 2004, 42 (2), 276–284. 10.1002/mrc.1334. 14745808

[ref30] Lorieau J. L.; McDermott A. E. Conformational Flexibility of a Microcrystalline Globular Protein: Order Parameters by Solid-State NMR Spectroscopy. J. Am. Chem. Soc. 2006, 128 (35), 11505–11512. 10.1021/ja062443u. 16939274

[ref31] Byeon I.-J. L.; Hou G.; Han Y.; Suiter C. L.; Ahn J.; Jung J.; Byeon C.-H.; Gronenborn A. M.; Polenova T. Motions on the Millisecond Time Scale and Multiple Conformations of HIV-1 Capsid Protein: Implications for Structural Polymorphism of CA Assemblies. J. Am. Chem. Soc. 2012, 134 (14), 6455–6466. 10.1021/ja300937v. 22428579 PMC3325613

[ref32] Taware P. P.; Jain M. G.; Raran-Kurussi S.; Agarwal V.; Madhu P. K.; Mote K. R. Measuring Dipolar Order Parameters in Nondeuterated Proteins Using Solid-State NMR at the Magic-Angle-Spinning Frequency of 100 kHz. J. Phys. Chem. Lett. 2023, 14 (15), 3627–3635. 10.1021/acs.jpclett.3c00492. 37026698

[ref33] Munowitz M. G.; Griffin R. G.; Bodenhausen G.; Huang T. H. Two-Dimensional Rotational Spin-Echo Nuclear Magnetic Resonance in Solids: Correlation of Chemical Shift and Dipolar Interactions. J. Am. Chem. Soc. 1981, 103 (10), 2529–2533. 10.1021/ja00400a007.

[ref34] Hong M.; Gross J. D.; Griffin R. G. Site-Resolved Determination of Peptide Torsion Angle φ from the Relative Orientations of Backbone N–H and C–H Bonds by Solid-State NMR. J. Phys. Chem. B 1997, 101 (30), 5869–5874. 10.1021/jp970887u.

[ref35] Hou G.; Byeon I.-J. L.; Ahn J.; Gronenborn A. M.; Polenova T. 1H–13C/1H–15N Heteronuclear Dipolar Recoupling by R-Symmetry Sequences Under Fast Magic Angle Spinning for Dynamics Analysis of Biological and Organic Solids. J. Am. Chem. Soc. 2011, 133 (46), 18646–18655. 10.1021/ja203771a. 21995349 PMC3218250

[ref36] Lu X.; Zhang H.; Lu M.; Vega A. J.; Hou G.; Polenova T. Improving Dipolar Recoupling for Site-Specific Structural and Dynamics Studies in Biosolids NMR: Windowed RN-Symmetry Sequences. Phys. Chem. Chem. Phys. 2016, 18 (5), 4035–4044. 10.1039/C5CP07818K. 26776070 PMC5240527

[ref37] Schneider R.; Seidel K.; Etzkorn M.; Lange A.; Becker S.; Baldus M. Probing Molecular Motion by Double-Quantum (13C,13C) Solid-State NMR Spectroscopy: Application to Ubiquitin. J. Am. Chem. Soc. 2010, 132 (1), 223–233. 10.1021/ja906283h. 20000710

[ref38] Liang L.; Ji Y.; Chen K.; Gao P.; Zhao Z.; Hou G. Solid-State NMR Dipolar and Chemical Shift Anisotropy Recoupling Techniques for Structural and Dynamical Studies in Biological Systems. Chem. Rev. 2022, 122 (10), 9880–9942. 10.1021/acs.chemrev.1c00779. 35006680

[ref39] Gullion T.; Schaefer J. Rotational-Echo Double-Resonance NMR. J. Magn. Reson. 1969 1989, 81 (1), 196–200. 10.1016/0022-2364(89)90280-1. 22152360

[ref40] Jain M. G.; Rajalakshmi G.; Agarwal V.; Madhu P. K.; Mote K. R. On the Direct Relation between REDOR and DIPSHIFT Experiments in Solid-State NMR. J. Magn. Reson. 2019, 308, 106563 10.1016/j.jmr.2019.07.050. 31353014

[ref41] Mueller K. T. Analytic Solutions for the Time Evolution of Dipolar-Dephasing NMR Signals. J. Magn. Reson. A 1995, 113 (1), 81–93. 10.1006/jmra.1995.1059.

[ref42] Shcherbakov A. A.; Hong M. Rapid Measurement of Long-Range Distances in Proteins by Multidimensional 13C–19F REDOR NMR under Fast Magic-Angle Spinning. J. Biomol. NMR 2018, 71 (1), 31–43. 10.1007/s10858-018-0187-0. 29785460 PMC6314655

[ref43] Gelenter M. D.; Chen K. J.; Hong M. Off-Resonance 13C–2H REDOR NMR for Site-Resolved Studies of Molecular Motion. J. Biomol. NMR 2021, 75 (8), 335–345. 10.1007/s10858-021-00377-7. 34342847 PMC8830769

[ref44] Kashefi M.; Malik N.; Struppe J. O.; Thompson L. K. Carbon-Nitrogen REDOR to Identify Ms-Timescale Mobility in Proteins. J. Magn. Reson. 2019, 305, 5–15. 10.1016/j.jmr.2019.05.008. 31158793 PMC6656615

[ref45] Bennett A. E.; Griffin R. G.; Ok J. H.; Vega S. Chemical Shift Correlation Spectroscopy in Rotating Solids: Radio Frequency-driven Dipolar Recoupling and Longitudinal Exchange. J. Chem. Phys. 1992, 96 (11), 8624–8627. 10.1063/1.462267.

[ref46] Abramov G.; Morag O.; Goldbourt A. Magic-Angle Spinning NMR of a Class I Filamentous Bacteriophage Virus. J. Phys. Chem. B 2011, 115 (31), 9671–9680. 10.1021/jp2040955. 21702439

[ref47] Bax A.; Freeman R.; Frenkiel T. A. An NMR Technique for Tracing out the Carbon Skeleton of an Organic Molecule. J. Am. Chem. Soc. 1981, 103 (8), 2102–2104. 10.1021/ja00398a044.

[ref48] Lesage A.; Auger C.; Caldarelli S.; Emsley L. Determination of Through-Bond Carbon–Carbon Connectivities in Solid-State NMR Using the INADEQUATE Experiment. J. Am. Chem. Soc. 1997, 119 (33), 7867–7868. 10.1021/ja971089k.

[ref49] Morag O.; Abramov G.; Goldbourt A. Similarities and Differences within Members of the Ff Family of Filamentous Bacteriophage Viruses. J. Phys. Chem. B 2011, 115 (51), 15370–15379. 10.1021/jp2079742. 22085310

[ref50] Abramov G.; Shaharabani R.; Morag O.; Avinery R.; Haimovich A.; Oz I.; Beck R.; Goldbourt A. Structural Effects of Single Mutations in a Filamentous Viral Capsid Across Multiple Length Scales. Biomacromolecules 2017, 18 (8), 2258–2266. 10.1021/acs.biomac.7b00125. 28657731

[ref51] Morcombe C. R.; Zilm K. W. Chemical Shift Referencing in MAS Solid State NMR. J. Magn. Reson. 2003, 162 (2), 479–486. 10.1016/S1090-7807(03)00082-X. 12810033

[ref52] Delaglio F.; Grzesiek S.; Vuister G. W.; Zhu G.; Pfeifer J.; Bax A. NMRPipe: A Multidimensional Spectral Processing System Based on UNIX Pipes. J. Biomol. NMR 1995, 6 (3), 277–293. 10.1007/BF00197809. 8520220

[ref53] Lee W.; Tonelli M.; Markley J. L. NMRFAM-SPARKY: Enhanced Software for Biomolecular NMR Spectroscopy. Bioinformatics 2015, 31 (8), 1325–1327. 10.1093/bioinformatics/btu830. 25505092 PMC4393527

[ref54] Lee W.; Rahimi M.; Lee Y.; Chiu A. POKY: A Software Suite for Multidimensional NMR and 3D Structure Calculation of Biomolecules. Bioinformatics 2021, 37 (18), 3041–3042. 10.1093/bioinformatics/btab180. 33715003 PMC8479676

[ref55] Aharoni T.; Goldbourt A. Rapid Automated Determination of Chemical Shift Anisotropy Values in the Carbonyl and Carboxyl Groups of Fd-Y21m Bacteriophage Using Solid State NMR. J. Biomol. NMR 2018, 72 (1), 55–67. 10.1007/s10858-018-0206-1. 30141148

[ref56] Bak M.; Rasmussen J. T.; Nielsen N. C. SIMPSON: A General Simulation Program for Solid-State NMR Spectroscopy. J. Magn. Reson. 2000, 147 (2), 296–330. 10.1006/jmre.2000.2179. 11097821

[ref57] Jaroniec C. P.; Tounge B. A.; Rienstra C. M.; Herzfeld J.; Griffin R. G. Recoupling of Heteronuclear Dipolar Interactions with Rotational-Echo Double-Resonance at High Magic-Angle Spinning Frequencies. J. Magn. Reson. 2000, 146 (1), 132–139. 10.1006/jmre.2000.2128. 10968966

[ref58] Berg J. M.; Tymoczko J. L.; Gatto G. J. Jr.; Stryer L. Biochemistry, 8 ed.W. H. Freeman & Company, 2015.

[ref59] Ladizhansky V.; Jaroniec C. P.; Diehl A.; Oschkinat H.; Griffin R. G. Measurement of Multiple ψ Torsion Angles in Uniformly 13C,15N-Labeled α-Spectrin SH3 Domain Using 3D 15N–13C–13C–15N MAS Dipolar-Chemical Shift Correlation Spectroscopy. J. Am. Chem. Soc. 2003, 125 (22), 6827–6833. 10.1021/ja029082c. 12769594

[ref60] Aharoni T.; Goldbourt A. Dynamics and Rigidity of an Intact Filamentous Bacteriophage Virus Probed by Magic Angle Spinning NMR. Chem. – Eur. J. 2018, 24 (35), 8737–8741. 10.1002/chem.201800532. 29660798

[ref61] Cross T. A.; Opella S. J. Protein Dynamics by Solid-State Nuclear Magnetic Resonance Spectroscopy: Peptide Backbone of the Coat Protein in Fd Bacteriophage. J. Mol. Biol. 1982, 159 (3), 543–549. 10.1016/0022-2836(82)90301-1. 7166755

[ref62] Colnago L. A.; Valentine K. G.; Opella S. J. Dynamics of Fd Coat Protein in the Bacteriophage. Biochemistry 1987, 26 (3), 847–854. 10.1021/bi00377a028. 3552033

[ref63] Lorieau J. L.; Day L. A.; McDermott A. E. Conformational Dynamics of an Intact Virus: Order Parameters for the Coat Protein of Pf1 Bacteriophage. Proc. Natl. Acad. Sci. U. S. A. 2008, 105 (30), 10366–10371. 10.1073/pnas.0800405105. 18653759 PMC2492469

[ref64] Franks W. T.; Zhou D. H.; Wylie B. J.; Money B. G.; Graesser D. T.; Frericks H. L.; Sahota G.; Rienstra C. M. Magic-Angle Spinning Solid-State NMR Spectroscopy of the Β1 Immunoglobulin Binding Domain of Protein G (GB1): 15N and 13C Chemical Shift Assignments and Conformational Analysis. J. Am. Chem. Soc. 2005, 127 (35), 12291–12305. 10.1021/ja044497e. 16131207

[ref65] Wylie B. J.; Franks W. T.; Rienstra C. M. Determinations of 15N Chemical Shift Anisotropy Magnitudes in a Uniformly 15N,13C-Labeled Microcrystalline Protein by Three-Dimensional Magic-Angle Spinning Nuclear Magnetic Resonance Spectroscopy. J. Phys. Chem. B 2006, 110 (22), 10926–10936. 10.1021/jp060507h. 16771346

[ref66] Wylie B. J.; Sperling L. J.; Frericks H. L.; Shah G. J.; Franks W. T.; Rienstra C. M. Chemical-Shift Anisotropy Measurements of Amide and Carbonyl Resonances in a Microcrystalline Protein with Slow Magic-Angle Spinning NMR Spectroscopy. J. Am. Chem. Soc. 2007, 129 (17), 5318–5319. 10.1021/ja0701199. 17425317

[ref67] Xu J.; Dayan N.; Goldbourt A.; Xiang Y. Cryo-Electron Microscopy Structure of the Filamentous Bacteriophage IKe. Proc. Natl. Acad. Sci. U. S. A. 2019, 116 (12), 5493–5498. 10.1073/pnas.1811929116. 30819888 PMC6431161

[ref68] Sergeyev I. V.; Bahri S.; Day L. A.; McDermott A. E. Pf1 Bacteriophage Hydration by Magic Angle Spinning Solid-State NMR. J. Chem. Phys. 2014, 141 (22), 22D533 10.1063/1.4903230. 25494804

[ref69] Marcovitz A.; Naftaly A.; Levy Y. Water Organization between Oppositely Charged Surfaces: Implications for Protein Sliding along DNA a). J. Chem. Phys. 2015, 142 (8), 085102 10.1063/1.4913370. 25725757

[ref70] Morag O.; Abramov G.; Goldbourt A. Complete Chemical Shift Assignment of the ssDNA in the Filamentous Bacteriophage Fd Reports on Its Conformation and on Its Interface with the Capsid Shell. J. Am. Chem. Soc. 2014, 136 (6), 2292–2301. 10.1021/ja412178n. 24447194

[ref71] Gall C. M.; Cross T. A.; DiVerdi J. A.; Opella S. J. Protein Dynamics by Solid-State NMR: Aromatic Rings of the Coat Protein in Fd Bacteriophage. Proc. Natl. Acad. Sci. U. S. A. 1982, 79 (1), 101–105. 10.1073/pnas.79.1.101. 6948294 PMC345669

[ref72] Li J.; Sae Her A.; Traaseth N. J. Site-Specific Resolution of Anionic Residues in Proteins Using Solid-State NMR Spectroscopy. J. Biomol. NMR 2020, 74 (6), 355–363. 10.1007/s10858-020-00323-z. 32514875 PMC7472563

[ref73] Yang J.; Tasayco M. L.; Polenova T. Magic Angle Spinning NMR Experiments for Structural Studies of Differentially Enriched Protein Interfaces and Protein Assemblies. J. Am. Chem. Soc. 2008, 130 (17), 5798–5807. 10.1021/ja711304e. 18393505

[ref74] Fang X.; Schmidt-Rohr K. Fate of the Amino Acid in Glucose–Glycine Melanoidins Investigated by Solid-State Nuclear Magnetic Resonance (NMR). J. Agric. Food Chem. 2009, 57 (22), 10701–10711. 10.1021/jf9020587. 19919118

[ref75] Schmidt-Rohr K.; Fritzsching K. J.; Liao S. Y.; Hong M. Spectral Editing of Two-Dimensional Magic-Angle-Spinning Solid-State NMR Spectra for Protein Resonance Assignment and Structure Determination. J. Biomol. NMR 2012, 54 (4), 343–353. 10.1007/s10858-012-9676-8. 23053913 PMC3656487

[ref76] Guo C.; Hou G.; Lu X.; Polenova T. Mapping Protein–Protein Interactions by Double-REDOR-Filtered Magic Angle Spinning NMR Spectroscopy. J. Biomol. NMR 2017, 67 (2), 95–108. 10.1007/s10858-016-0086-1. 28120201 PMC6258002

[ref77] Blümich B. Contrast in Solid-State NMR Imaging Part IIa: Basic Filters. Concepts Magn. Resons. 1999, 11 (2), 71–87. 10.1002/(SICI)1099-0534(1999)11:2<71::AID-CMR2>3.0.CO;2-3.

