# Supplementary material for: Dynamics in the Intact fd Bacteriophage Revealed by Pseudo 3D REDOR-Based Magic Angle Spinning NMR
Source: JACS Au. 2024 Aug 26;4(9):3619–28. doi: 10.1021/jacsau.4c00549 (PMC11423308; doi:10.1021/jacsau.4c00549)
Supplement: Supplementary file 1 — au4c00549_si_001.pdf [file au4c00549_si_001.pdf]

Supporting Information

Dynamics in the Intact fd Bacteriophage Revealed by Pseudo 3D REDOR-Based Magic Angle Spinning NMR

Orr Simon Lusky[1], Dvir Sherer[1], Amir Goldbourt[1,2*]

[1]*School of Chemistry, Faculty of Exact sciences, Tel Aviv University, Tel Aviv 6997801, Israel.*
[2]*The Center for Physics and Chemistry of Living Systems, Tel Aviv University, Tel Aviv 6997801, Israel.*

*amirgo@tauex.tau.ac.il

S1 Experimental parameters

We conducted three types of experiments, all involving a REDOR dephasing block, and three different mixing schemes: DARR, RFDR and INADEQUATE. In DARR and RFDR the mixing scheme follows the REDOR dephasing, whereas the INADEQUATE mixing precedes the dephasing. All experiments share these parameters:

Field [T]	14.1T
Probe	4mm E-free
MAS rate (ν_R) [kHz]	14.0
Carrier frequency [ppm]	98.0
^{1}H 90° pulse duration [µs]	2.60
^{13}C 90° pulse duration [µs]	4.50
^{15}N 180° pulse duration [µs]	10.25
CP power (ν_H) [kHz]	78.5
CP power (ν_C) [kHz]	55.5
CP contact time [ms]	1.50
^{1}H decoupling power [kHz]	85
swf-tppm decoupling pulse duration [µs]	6.0
Relaxation delay (~5T_1) [s]	3.0
Set temperature at the controller [°C]	-15
Estimated actual temperature* [°C]	11±1
**Processing parameters F1/F2**	
Processing software	NMRPipe
Zero fill (t1/t2)	2048/8192
Apodisation (F1/F2)	Squared cosine/Cosine

*The actual temperature was estimated using the chemical shift of water in the sample.

DARR-REDOR experimental parameters

Dephasing times [µs]	429, 571, 857, 1000, 1143, 1286, 1571
Acquisition mode in indirect dimension	States
Acquisition points (t1/t2)	720/4990
Acquisition times (t1/t2) [ms]	9.00/25.00
Spectral width F1/F2 [kHz]	40/100

^{1}H power during DARR [kHz]	14
DARR mixing time [ms]	5.00
Scans	16
Experimental time (S_0 and S) [hours]	~19
Analysis software	SPARKY 3.134.

RFDR-REDOR experimental parameters

Dephasing times [μs]	429, 857, 1000, 1143, 1286, 1429, 1571
Acquisition mode in indirect dimension	TPPI
Acquisition points (t1/t2)	720/4990
Acquisition times (t1/t2) [ms]	9.00/25.00
Spectral width F1/F2 [kHz]	40/100
RFDR mixing time [ms]	6.00
RFDR ^{13}C 180° pulse length [μs]	9.00
^{1}H LG decoupling power [kHz]	85
^{1}H LG offset [kHz]	60.1
Scans	32
Experimental time (S_0 and S) [hours]	~38.5
Analysis software	SPARKY 3.134

INADEQUATE-REDOR experimental parameters

Dephasing times [μs]	429, 571, 714, 1143, 1286, 1429, 1571, 1714
Acquisition mode in indirect dimension	States
Acquisition points (t1/t2)	450/3988
Acquisition time (t1/t2) [ms]	3.75/20.00
Spectral Width F1/F2 [kHz]	60/100
τ† [ms]	3.50
z-filter duration [μs]	71.43
Scans	32
Experimental time (S_0 and S) [hours]	~24
Analysis software	POKY[2]

† See Figure S1

The noise level was estimated by the noise calculation function of SPARKY. The noise was calculated as the average of 10 estimations.

The phase scheme of the dephasing pulses of the REDOR block was XY8.[3]

Complete phase cycles are shown explicitly in Figure S1.

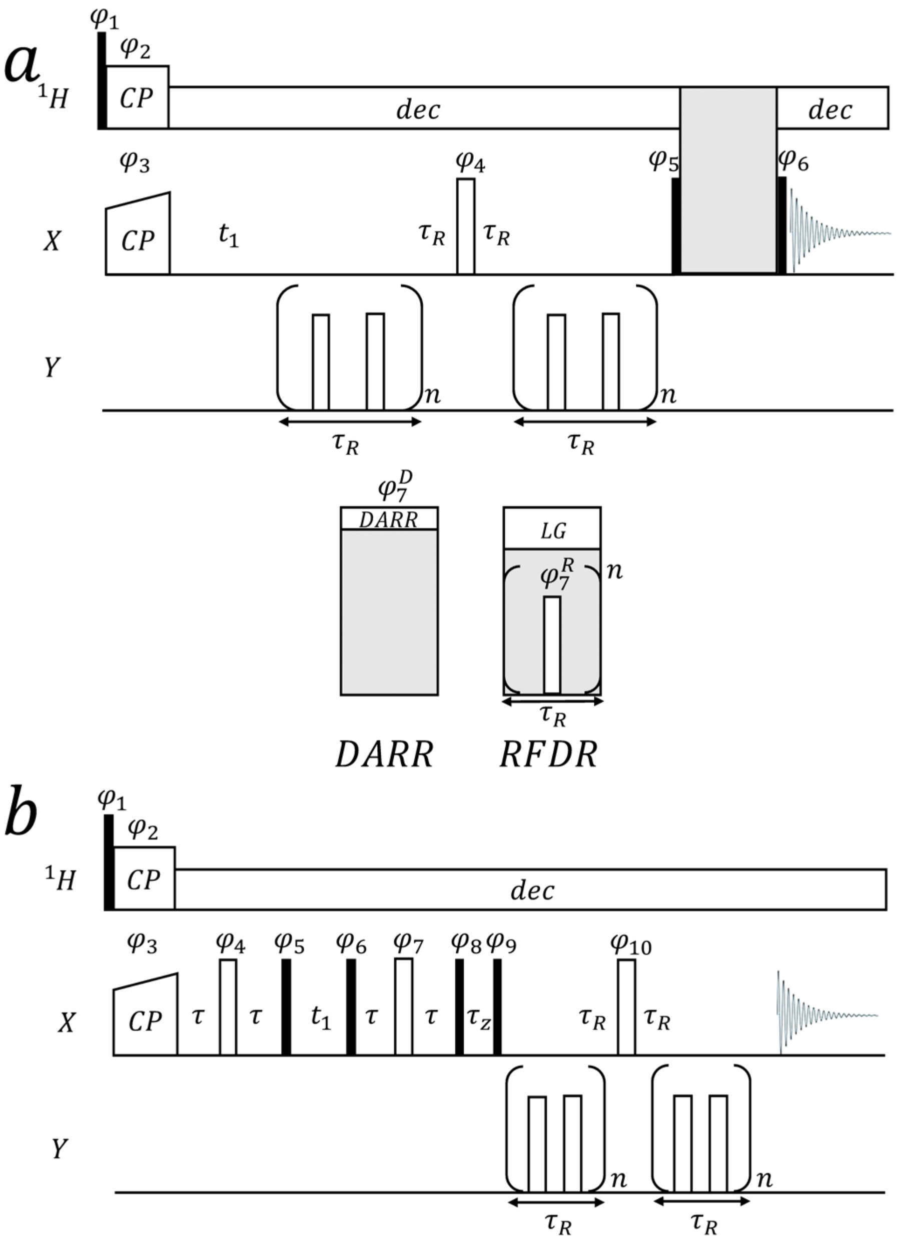

Figure S1. Pulse sequences for the measurement of C-N dipolar coupling constants. Black and white rectangles represent 90° and 180° pulses, respectively. CP stands for Cross Polarisation block, and 'dec' stands for ^{1}H decoupling. τ_R is the MAS rotor period. (a) X{Y}-X REDOR-DARR and REDOR-RFDR pulse sequences. The two applied schemes are identical with the exception of the mixing block represented by the grey rectangle. For DARR mixing the ^{1}H radio-frequency irradiation strength ν_1 is applied during mixing at the rotary resonance condition $\nu_1 = \tau_R^{-1}$. For RFDR, rotor-synchronous 180° pulses are applied on the X channel (^{13}C in our experiments) and a homonuclear ^{1}H Lee-Goldburg (LG) decoupling is employed. τ_R is the MAS rotor period. $\varphi_1 = \{90°, 270°\}, \varphi_2 = \varphi_7^D = \{0°\}, \varphi_3 = \{90°\}, \varphi_4 = \{90°\}_2\ \{180°\}_2, \varphi_5 = \{0°\}_4\ \{180°\}_4, \varphi_6 = \{90°\}$. For the RFDR pulses, $\varphi_7^R = XY - 8$ was employed. (b) X-X{Y} INADEQUATE-REDOR: $\varphi_1 = \{90°, 270°\}$, $\varphi_2 = \varphi_6 = \varphi_8 = \varphi_9 = \{0°\}, \varphi_3 = \{90°\}_2\ \{180°\}_2\ \{270°\}_2\ \{0°\}_2, \varphi_4 = \varphi_5 = \{0°\}_2\ \{90°\}_2\ \{180°\}_2\ \{270°\}_2, \varphi_7 = \{90°\}_{16}\ \{270°\}_{16}, \varphi_{10} = \{90°\}$.

S2 – Matlab scripts for fitting the REDOR curves

The Matlab script I is used to analyse the results from the 'Cross Peaks Analysis Tool' of RAVEN.[4] The input is a string array named 'results'. The required format for 'n' crosspeaks with 'k' dephasing times is

$Crosspeak\ identifier$	δ_1	δ_2	$S(\tau_1)$	$S_0(\tau_1)$	$S(\tau_2)$	$S_0(\tau_2)$	$\cdots$	$S(\tau_k)$	$S_0(\tau_k)$
$Crosspeak\ \#1$	$\delta_{1,1}$	$\delta_{1,2}$	$intensity$	$intensity$	$intensity$	$intensity$	$\cdots$	$intensi$[illegible]y	$intensity$
$Crosspeak\ \#2$	$\delta_{2,1}$	$\delta_{2,2}$	$intensity$	$intensity$	$intensity$	$intensity$	$\cdots$	$intensity$	$intensity$
$\vdots$	$\vdots$	$\vdots$	$intensity$	$intensity$	$intensity$	$intensity$	$\cdots$	$intensity$	$intensity$
$Crosspeak\ \#n$	$\delta_{n,1}$	$\delta_{n,2}$	$intensity$	$intensity$	$intensity$	$intensity$	$\cdots$	$intensity$	$intensity$
			$noise$	$noise$	$noise$	$noise$	$\cdots$	$noise$	$noise$

The dephasing times τ_j are in units of μs.

An example of actual data:

			428.6	428.6	571.4	571.4	857.1	857.1	1000	1000	1142.9	1142.9	1285.7	1285.7	1571.4	1571.4
K8Ca-Cb	59.81	32.79	13017212	14715122	11799954	14743988	9789653	13709820	8384717	15047111	6578601	13876720	5434314	13570522	3659389	12436655
K8Ca-Cg	59.93	25	3099990	3658567	2426522	3076466	2343983	3244493	1783701	3460326	1288482	3093055	1218291	2791504	376956.2	2825963
A10Ca-Cb	54.94	20.8	13652908	15166148	11295424	14656649	8781171	13244710	8132726	13724887	6385731	13374704	6363556	12789240	4084359	11535716
			204400	209300	198100	190500	192600	178700	206000	179400	206600	203200	192600	185500	182700	177500

The script calculates 1-S_d for each data point, and then calculates the χ^2_{red} value for various dipolar coupling constants according to

$$\chi^2_{red}(d) = \frac{1}{N-1}\sum_{i}^{N}\frac{(O_i^2 - F_i^2)}{\sigma_i^2},$$

with 'd' being a specific calculated dipolar coupling constant, 'N' being the number of points in the REDOR dimension, 'O' being the observed value $1 - S_d(\tau_n)$, 'F' being the theoretical value of $1 - S_d(\tau_n)$ corresponding to the specific d according to the universal curve, and 'σ' is the error, calculated by

$$\sigma_i = |S_{d,i}|\sqrt{\left(\frac{n_{s,i}}{S_i}\right)^2 + \left(\frac{n_{s_0,i}}{S_{0,i}}\right)^2},$$

with $n_{s,i}$ and $n_{s_0,i}$ being the estimated noise level of the 'S' and 'S_0' spectra respectively.

The effective dipolar coupling constant of a specific spin, d, is the one corresponding to the minimum χ^2_{red} value ($\chi^2_{red,min}$). The error of the fit was calculated according to the span of dipolar coupling constants that fulfil $\chi^2_{red}(d) = 2 \cdot \chi^2_{red,min}(d_{eff})$.

Matlab script I:

```
a = size(results);

%Creating a matrix of dephasing times in units of sec
j = 4;
deph = [];
data = ["crosspeak"];
while j<a(2)
```

```
    deph = [deph, results(1,j)];
    data = [data, append(results(1,j)," µs")];
    j = j+2;
end
deph = 10^-6*str2double(deph);

%Set the screened dipolar constants
res = 1; %The resolution in the screened dipolar constants in Hz
dip = [5:res:2000]; %array of wanted dipolar constants range in Hz
tau = [0:0.0010:max(deph)*10^3]; %time in ms

%Creating a string array of crosspeaks, sd and error matrix
m = 2;
error = [];
while m<a(1)
        temp = results(m,1);
        line = [temp];
        linen = [];
        j = 4;
        while j<a(2)
            s = str2double(results(m,j));
            n = str2double(results(a(1),j));
            s0 = str2double(results(m,j+1));
            n0 = str2double(results(a(1),j+1));
            sd = 1-s/s0;
            noise = abs(s/s0)*sqrt((n0/s0)^2+(n/s)^2);
            line = [line, sd];
            linen = [linen,noise];
            j = j+2;
        end
        data = [data; line];
        error = [error;linen];
        m = m+1;
end

%Calculate χ^2_red for each crosspeak and determine the dipole constant
coef = 0.25*sqrt(2)*pi();
[b,~] = size(data);
[~,c] = size(deph);
[~,d] = size(dip);
i = 1;
while i<d+1
    column = [(dip(i))];
    m = 2;
    while m<b+1
        j = 2;
        r = 0;
        while j<c+2
            sd = str2double(data(m,j));
            lam = sqrt(2)*deph(j-1)*dip(i);
            SD = 1-coef*besselj(0.25,lam)*besselj(-0.25,lam);
            temperr = error(m-1,j-1);
            r = r + ((SD-sd)/temperr)^2;
            j = j+1;
        end
```

```
        r = r/(c-1);
        column = [column;r];
        m = m+1;
    end
    data = [data,column];
    i = i+1;
end

%Calculate the error in the effective dipolar interaction
RMSDtable = str2double(data(:,(c+2):(c+d+1)));
m = 2;
column1 = ["Dipole constant in Hz"];
column2 = ["χ^2_red"];
column3 = ["Error"];

while m<b+1
    line = RMSDtable(m,:);
    [M,I] = min(line);

    err = 2*M;
    p = I;
    while line(p)<=err
        p = p-1;
        if p==0
            leftedge = dip(1);
            break
        end
        leftedge = dip(p+1);
    end

    p = I;
    while line(p)<=err
        p = p+1;
        if p>d
            rightedge = dip(1);
            break
        end
        rightedge = dip(p-1);
    end

    if (rightedge - dip(I)) == 0
        plus = append("^{+",num2str(res),"}");
    else
        plus = append("^{+",num2str(rightedge - dip(I)),"}");
    end
    if (dip(I) - leftedge) == 0
        minus = append("_{-",num2str(res),"}");
    else
        minus = append("_{-",num2str(dip(I) - leftedge),"}");
    end
    column3 = [column3;append(plus,minus)];

    M = num2str(M);
    I = num2str(dip(I));
    column1 = [column1; append(I)];
```

```
    column2 = [column2; M];
    m = m+1;
end
data = [data, column1, column2, column3];

%Plot the curve with the data points on it for each crosspeak
    f1 = figure;
    figure(f1);
    t=tiledlayout('flow');
    nexttile
    m = 2;
    while m<b+1
        dipolar = str2num(data(m,c+d+2));
        lam = sqrt(2)*dipolar.*tau*10^-3;
        sd = 1-coef*besselj(0.25,lam).*besselj(-0.25,lam);
        plot(tau,sd,'LineWidth', 1.5);
        x = deph*10^3;
        hold on
        j = 2;
        y = [];
        while j<(c+2)
            y = [y,str2double(data(m,j))];
            j = j+1;
        end
        temperr = error(m-1,:);
        errorbar(x,y,temperr, temperr,'o');
        set(gca,'FontSize',15);
        dipolar = num2str(dipolar);
        [~,f] = size(data);
        s = append(data(m,1),'; ', dipolar, data(m,f), ' ', 'Hz');
        title(s);
        xlabel("dephasing time [ms]", 'FontSize', 15);
        ylabel("1-S/S_0", 'FontSize', 15);
        if m==b(1)
            hold off
        else
            nexttile
        end
        m = m+1;
    end

%Plot χ^2_red(d) for each crosspeak
f2 = figure;
figure(f2);
t2 = tiledlayout('flow');
nexttile
m = 2;
while m<b+1
    i = c+2;
    y = [];
    while i<(d+c+2)
        y = [y, str2num(data(m,i))];
        i = i+1;
    end
    scatter(dip,y, 'LineWidth', 1.5);
```

```
    title(data(m,1));
    xlabel("Dipolar constant in Hz", 'FontSize', 15);
    ylabel("χ^2_r_e_d", 'FontSize', 15);
    if m==b(1)
        hold off
    else
        nexttile
    end
    m = m+1;
end
```

Matlab script II: This script is used in order to convert the ‘matches’ output file of RAVEN into a new list in the required format for the ‘Cross Peaks Analysis Tool’ of RAVEN.

```
%Takes 'matches' file from Raven and turns it into a list of unambiguous crosspeaks
as 'new.list', that can be used for the crosspeak analysis tool of Raven.

A = readlines('matches.txt');
k = size(A);
i = 2;
j = 2;
c = [];
while i<k(1)
    temp = [];
    if A(i) ~= ""
        j = i;
        while A(j) ~= ""
            temp = [temp; A(j)];
            j = j+1;
        end
    end
    [token1, remain] = strtok(temp);
    [token, remain] = strtok(remain);
    if all(token==token(1))
        c = [c;token];
    end
    i=j+1;
end

t = size(c);
i = 1;
d = ["Assignment" "w1" "w2"];
while i<t(1)+1
    [name1,remain] = strtok(c(i),'(');
    [cs1,remain] = strtok(remain,'-');
    [name2,cs2] = strtok(remain,'(');
    name = append(name1,name2);
    w1 = erase(cs1,'(');
    w1 = erase(w1,')');
    w2 = erase(cs2,'(');
    w2 = erase(w2,')');
    i = i+1;
```

```
    d = [d;name, w1, w2];
end

fileID = fopen('new.list', 'w');
fprintf(fileID, '%s \n', 'CC');
fprintf(fileID,'%s %s %s \n',d');
fclose(fileID);
```

Both scripts were written on version R2021b of Matlab by MathWorks Inc.

S3 – DQ extension for RAVEN and POKY

Both RAVEN and POKY do not support DQ data. In order to make the analysis process for the INADEQUATE-REDOR p3D experiment automated (or in general use RAVEN or POKY for DQ data), we wrote a Python script that manipulates the DQ data to fit the necessary formats for the two programmes.

DQ data in RAVEN: The script creates a new sequence file with additional residues that allows the representation of DQ data by duplicating the original sequence. Since RAVEN does not support assignment tables with resonances that do not correspond to predefined conventional atoms as they appear in the BMBR, the modified table labels all possible combinations of bonded atoms, which can potentially be obtained from J-based DQ correlations, as single spins. This is demonstrated in Figure S3.1.

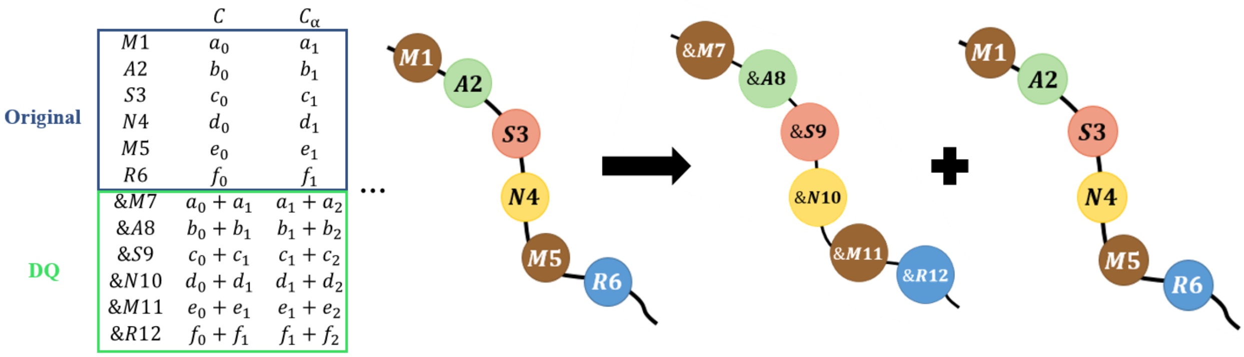

Figure S3.1. Illustration of the conversion process on a six-residue-long sequence. The left panel demonstrates the sequence duplication. The script denotes the fictitious residues with '&' and calculates the DQ resonances based on the original assignment table. The subscript '2' in a_2, b_2, etc. corresponds to C_β. Additional a_k, b_k, etc. are generated according to the length of the particular amino acids.

The automated assignment process done by RAVEN yields a 'matches' file that includes all possible assignment options for resonances with the addition of the DQ data. The script then converts the fictitious labels ("new" amino acids and resonances) into the proper labels. In the process it filters out assignment options that are either impossible in J-based INADEQUATE (inter-residues contacts) or improbable ($^2J_{CC}$ and higher contacts). The user can also specify the ^{13}C isotopic labelling scheme that was used for the sample preparation, including probabilities resulting from scrambling. The script filters out improbable assignment options according to a scrambling threshold (joint

probability for an assigned spin-pair) selected by the user. The result is a filtered 'match' file with DQ data. This process is illustrated in Figure S3.2.

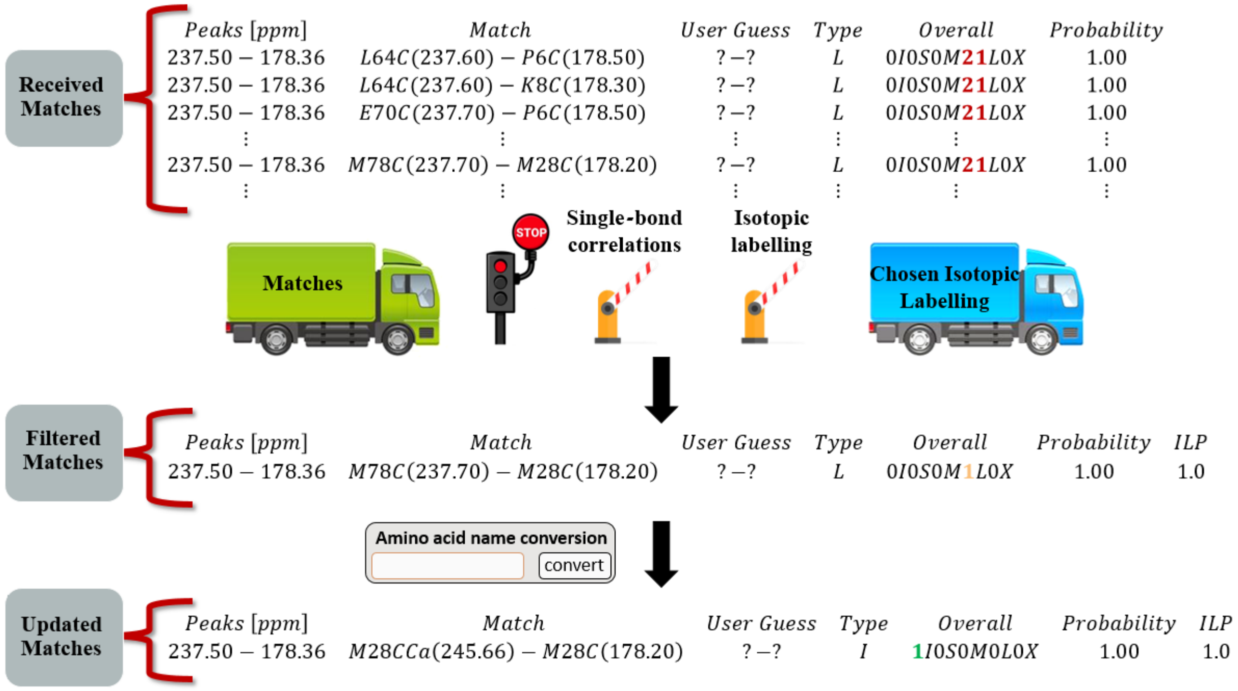

Figure S3.2. Illustration of the conversion of fictitious-DQ notation of the RAVEN 'matches' file into an explicit DQ peak list. The First block contains all possible assignment options for crosspeaks in the 50-residues long coat protein from the fd-Y21M phage coat protein. Matches are filtered by eliminating the improbable assignments based on the labelling scheme and on chemical bonds. Then, the labels of the amino acids are converted into their proper labels. Here M28CCa(245.66) represents a double-quantum peak correlation of methionine #28 with the sum of C (carbonyl) and Cα in the indirect dimension at 245.66 ppm, and the carbonyl resonance at 178.2 ppm.

The python script supports two additional RAVEN features that are non-specific to the DQ data. (i) It can convert 'matches' file into the 'Cross Peak Analysis Tool' format of RAVEN, and (ii) it can compare the 'matches' file with the peak list to identify the crosspeaks that were not assigned by RAVEN.

DQ data in POKY: Another function in the script creates a POKY-formatted assignment table with DQ resonances. The result can be used to analyse INADEQUATE spectra in POKY, as demonstrated in Figure S3.3.

An independent script suitable for POKY alone has also been written and is available in the GitHub repository.

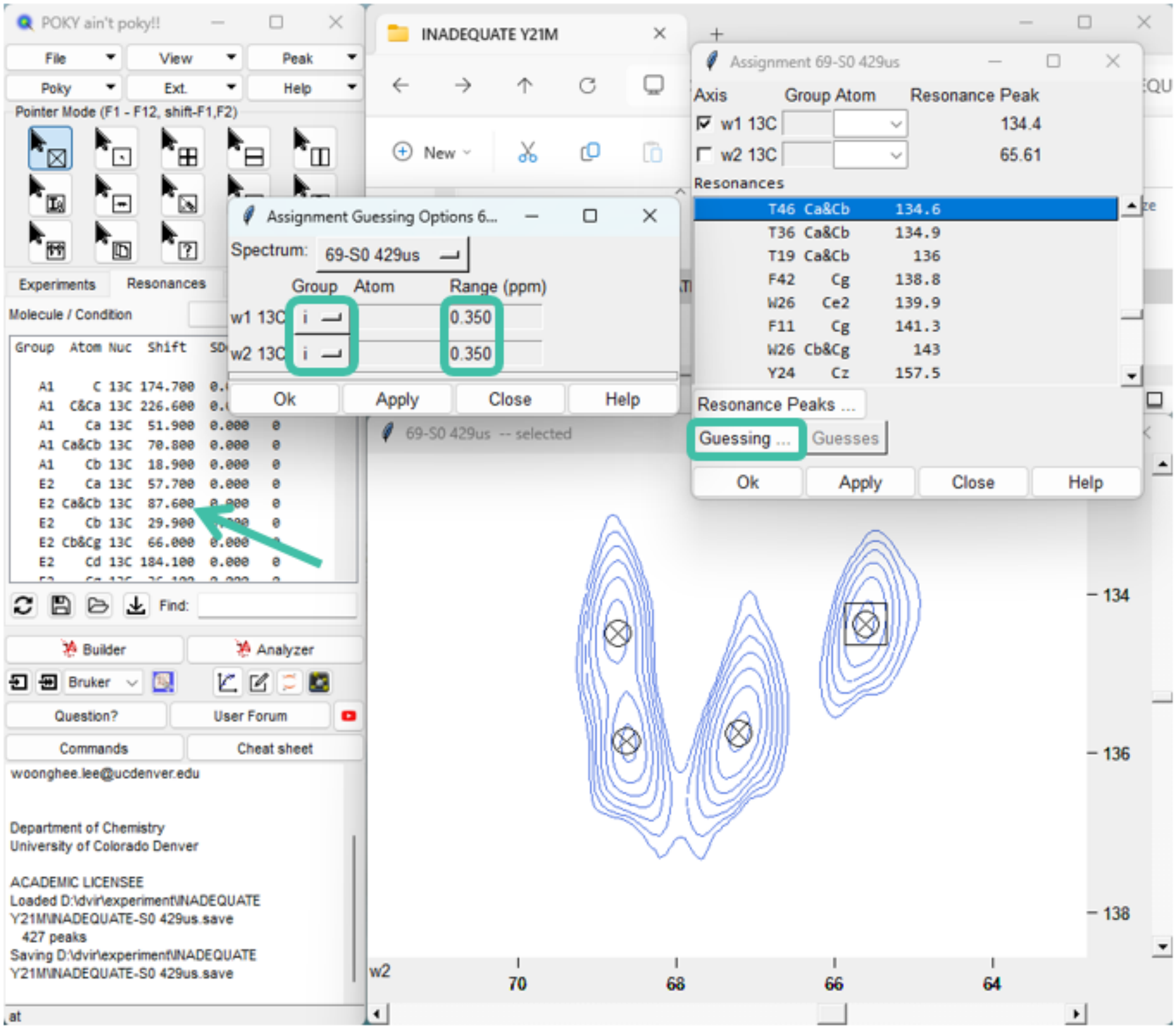

Figure S3.3. Screenshots of the POKY software interface. The spectrum is from the ‘S_0’ INADEQUATE-REDOR experiment collected with a REDOR dephasing time of 429μs, conducted on the intact fd-Y21M phage. The arrow points at the modified assignment table that supports the DQ data. The green rectangles show the function in POKY that guesses the assignment of crosspeaks according to the modified assignment table.

The full script, along with tutorial videos and a manual can be found in GitHub, https://github.com/amirgoldbourt/DQ-extension-for-RAVEN-and-POKY.

S4 – Validity of linear prediction and the universal REDOR curve approach in the analysis of p3D experiments

Validity of linear prediction: Linear prediction is used in order to increase the resolution in the indirect dimension by reducing the linewidths thereby improving sensitivity.[5] The linear prediction was needed for the INADEQUATE and RFDR based experiments, and it allowed the analysis of more residues. In order to ensure that the linear prediction does not change the results

(quantification of the REDOR curve), we calculated 1-S_d for crosspeaks that were non-ambiguous before the linear prediction and compared the results to those obtained from a spectrum with linear prediction.

Figure S4.1 shows the build-up curves for three crosspeaks with and without linear prediction as an example. The results of the fit are summarised in table S4 and show that the difference is smaller than the experimental errors, thus validating the usage of linear prediction.

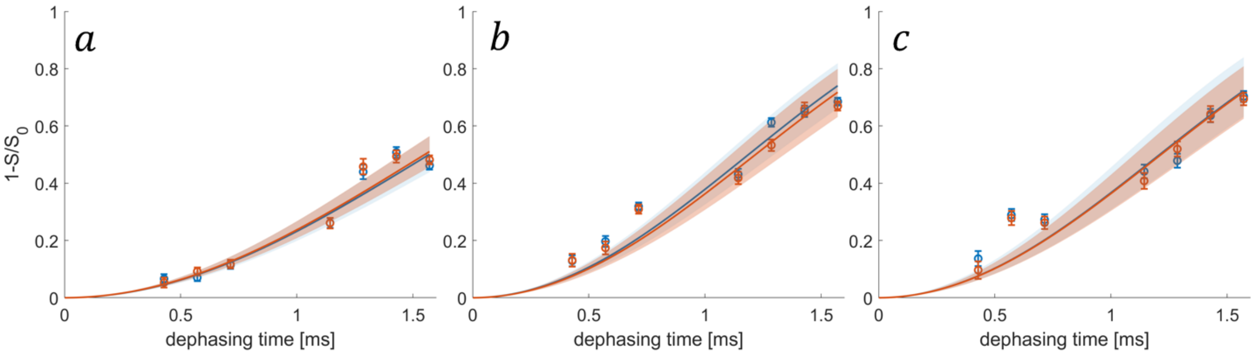

Figure S4.1. INADEQUTE-REDOR build up curves of three crosspeaks: (a) Q15 Cγ&Cδ-Cδ, (b) T46 Cα&Cβ-Cα, (c) T19 Cα&Cβ-Cα with linear prediction (blue) and without linear prediction (orange). The experimental data is shown in circles along with the errors. The curves represent the best fit to the universal REDOR curve. The shaded areas correspond to the level of confidence derived by the errors summarised in table S4.1.

Crosspeak	'd_{eff}' with linear prediction	'd_{eff}' without linear prediction
Q15 Cγ&Cδ-Cδ	488^{+39}_{-39}	494^{+34}_{-34}
T46 Cα&Cβ-Cα	645^{+59}_{-54}	629^{+60}_{-58}
T19 Cα&Cβ-Cα	633^{+77}_{-72}	630^{+66}_{-63}

Table S4.1. Effective dipolar coupling constants 'd' calculated by fits to the universal REDOR curve (in units of Hz) for three different crosspeaks in INADEQUATE-REDOR experiments with and without linear prediction.

Validity of the universal REDOR curve: The fit to the universal REDOR curves assumes an isolated two-spin system. In order to validate this assumption, we used the NMR simulation package SIMPSON[6] and its original REDOR script. First, we simulated a three-spin system consisting of one ^{13}C spin and two ^{15}N spins (CN_2). The strength of the heteronuclear dipolar coupling constants were chosen to be 1005Hz and 200Hz, derived from the typical distances between Cα and the two N spins of the same the adjacent amino acid. Figure S4.2a shows the simulated build-up curve along the best fit. We also simulated a four-spin system with three ^{13}C spins (representing C, Cα and Cβ) and one ^{15}N (C_3N). The homonuclear dipolar interactions Cα-C and Cα-Cβ were chosen to be 2250Hz (corresponding to a distance of 1.5Å), and the heteronuclear Cα-N dipolar interaction was set to 1005Hz. Figure S4.2b shows the simulated build-up curve with the fit. In both simulations an error of 5% was added to the data points in order to resemble experimental conditions.

For the CN_2 system the best fit yielded a result of $d_{eff} = 1007Hz$, a deviation of 2Hz. The C_3N system yielded an effective dipolar coupling constant of $d_{eff} = 1099Hz$, a deviation of 4Hz. These deviations are both smaller than the experimental errors, thus validating the assumptions.

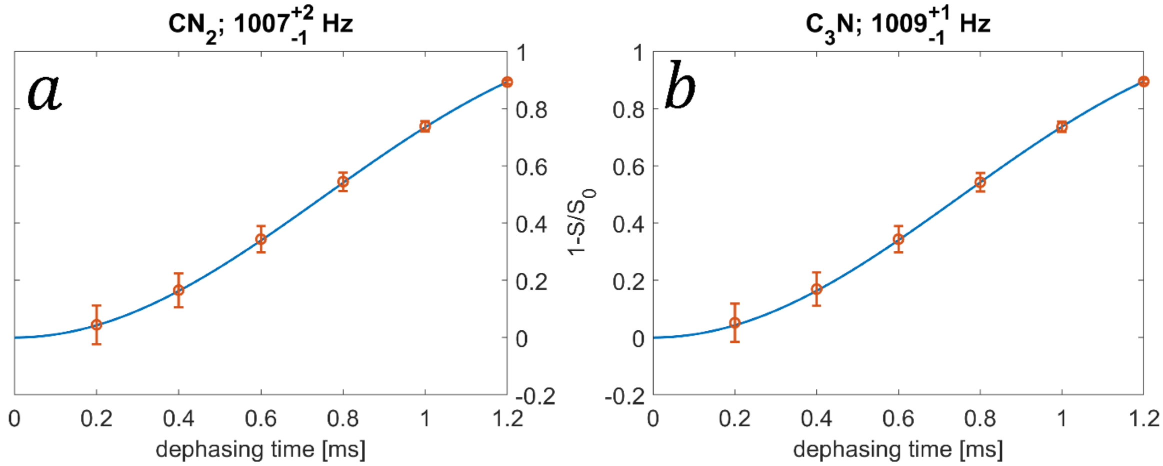

Figure S4.2. Build up curves of simulated systems (orange) along with the best fit to the universal REDOR curve (blue). (a) A system of Cα with two ^{15}N spins. The dipolar coupling constants were 1005Hz and 200Hz, corresponding to typical values in the backbone of proteins. (b) A system of C, Cα and Cβ spins along with a single ^{15}N spin attached to the Cα. The homonuclear and heteronuclear dipolar coupling constants were 2250Hz and 1005Hz, respectively.

S5 – Detailed example of the data analysis procedure

The general steps for data analysis are as follows:

- The spectra are recorded, Fourier transformed, and the required crosspeaks are peak-picked and recorded. The crosspeaks are then assigned automatically by RAVEN according to the assignment table.
- The RAVEN 'matches' file is converted into the required format (using the Matlab script I in S2) for the 'Cross Peak Analysis Tool' of RAVEN.
- The assigned peak list is uploaded onto the 'Cross Peak Analysis Tool' of RAVEN along with all the UCSF files (the format is generated by the nmrPipe 'ucsfdata' tool) corresponding to the p3D experiments.
- The build-up curves are saved and uploaded into Matlab (using script II) as a string array that includes the noise level of each experiment.
- The build-up curves are created along with a χ^2_{red} plot.

Figure S5 illustrates the stages of data analysis on the P6Cδ-Cγ crosspeak detected in the REDOR-DARR experiments.

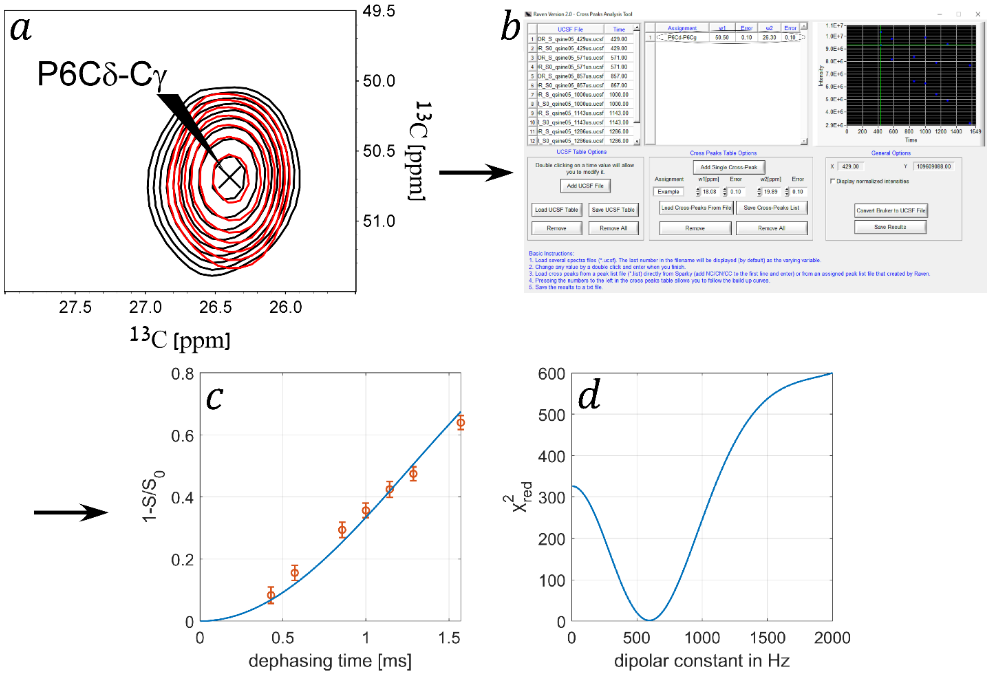

Figure S5. An illustration of the data analysis process. (a) The crosspeak P6Cδ-Cγ is identified in the DARR-REDOR spectra. The black and RED are the 'S_0' and 'S' spectra respectively acquired with a REDOR dephasing time of 857μs. Contour levels are drawn at multiples of 1.2 with the lowest matching an SNR of 6. (b) A screenshot of RAVEN Cross Peak Analysis Tool. For clarity, only the data of the crosspeak in (a) appear in the panel. (c) A REDOR build-up curve created by calculating 1-S_d using the results from (b) and the Matlab script I. The data points (1-S_d) are shown in orange with the errors, and the best fit curve is shown in blue. The fit yielded $d_{eff} = 595^{+28}_{-27}$. (d) χ^2_{red} plot for all screened dipolar coupling constants. Here $\chi^2_{red,min}\left(d_{eff} = 595Hz\right) = 1.66$.

The best fit yielded a result of $d_{eff} = 595^{+28}_{-27}$, which corresponds to an order parameter of $0.59^{+0.03}_{-0.03}$. In total, three crosspeaks involving the dephasing of P6Cδ were recorded – one in each mixing scheme. After analysing all three crosspeaks, the average order parameter for the Cδ-N bond was measured to be $0.58^{+0.03}_{-0.03}$.

S6 – Full results of dephasing curves

Table S6.1: Backbone Cα-N (and Cδ-N in P6) order parameters for the fd-Y21M phage coat protein. A dash represents a residue without data. The rigid limit is $d = 1005Hz$. The total amount of observed crosspeaks is indicated, along with 'D', 'R' and 'I' standing for the mixing scheme in which they appeared (D=DARR, R=RFDR, I=INADEQUATE) and the number of crosspeaks in each of the experiments (e.g. 3R: 3 peaks observed in REDOR-RFDR). Each pair of A7 and A35, D12 and L14, and I32 and I39 also has an addition of two ambiguous crosspeaks in both DARR and RFDR mixings that were analysed.

Residue	Information on resolved crosspeaks	Order parameter	Amplitude (Motion on a cone) (°)	Amplitude (Motion in a cone) (°)
A1	1 (R)	$0.16^{+0.02}_{-0.03}$	$48.4^{+1.2}_{-1.4}$	$75.2^{+2.0}_{-2.2}$
D2	1 (R)	$0.22^{+0.04}_{-0.06}$	$46.2^{+1.7}_{-2.1}$	$70.8^{+3.3}_{-4.1}$

G3	2 (R,I)	$0.26^{+0.04}_{-0.06}$	$44.4^{+1.7}_{-2.2}$	$67.5^{+3.1}_{-4.0}$
D4	2 (R)	$0.36^{+0.02}_{-0.05}$	$40.6^{+0.9}_{-1.0}$	$60.7^{+1.6}_{-1.7}$
D5	6 (D, 3R, 2I)	$0.50^{+0.03}_{-0.03}$	$35.2^{+1.2}_{-1.3}$	$51.7^{+2.0}_{-2.1}$
P6 Cα	5 (D, 3R, I)	$0.64^{+0.04}_{-0.04}$	$29.5^{+1.7}_{-1.6}$	$42.7^{+2.6}_{-2.4}$
P6 Cδ	3 (D, R, I)	$0.58^{+0.03}_{-0.03}$	$31.8^{+0.5}_{-0.4}$	$46.3^{+1.1}_{-1.1}$
A7	3 (D, R, I)	$0.67^{+0.03}_{-0.03}$	$27.9^{+0.9}_{-0.9}$	$40.3^{+2.1}_{-2.2}$
K8	7 (3D, 3R, I)	$0.69^{+0.02}_{-0.02}$	$26.8^{+1.0}_{-0.9}$	$38.7^{+1.6}_{-1.4}$
A9	-			
A10	4 (D, 2R, I)	$0.66^{+0.03}_{-0.03}$	$28.3^{+1.4}_{-1.4}$	$41.0^{+2.2}_{-2.1}$
F11	5 (2D, 2R, I)	$0.66^{+0.05}_{-0.04}$	$28.2^{+2.2}_{-1.9}$	$40.8^{+3.3}_{-2.8}$
D12	4 (2D, R, I)	$0.64^{+0.04}_{-0.03}$	$29.4^{+1.1}_{-1.1}$	$42.7^{+2.5}_{-2.4}$
S13	-			
L14	1 (I)	$0.64^{+0.04}_{-0.04}$	$29.2^{+1.2}_{-1.1}$	$42.3^{+2.6}_{-2.5}$
Q15	3 (2D, I)	$0.65^{+0.03}_{-0.03}$	$29.0^{+1.6}_{-1.5}$	$42.1^{+2.4}_{-2.3}$
A16	1 (D)	$0.66^{+0.05}_{-0.05}$	$28.3^{+2.5}_{-2.4}$	$41.0^{+3.8}_{-3.7}$
S17	-			
A18	1 (R)	$0.70^{+0.04}_{-0.04}$	$26.4^{+2.0}_{-1.9}$	$38.1^{+3.0}_{-2.9}$
T19	5 (D, 2R, 2I)	$0.63^{+0.03}_{-0.03}$	$29.9^{+1.2}_{-1.1}$	$43.4^{+1.8}_{-1.8}$
E20	4 (D, 2R, I)	$0.63^{+0.04}_{-0.04}$	$29.6^{+1.9}_{-1.7}$	$43.0^{+2.9}_{-2.6}$
M21	-			
I22	5 (2D, 2R, I)	$0.66^{+0.05}_{-0.04}$	$28.3^{+2.1}_{-2.0}$	$40.9^{+3.3}_{-3.0}$
G23	2 (D, I)	$0.69^{+0.04}_{-0.04}$	$27.1^{+2.1}_{-2.0}$	$39.1^{+3.2}_{-3.0}$
Y24	-			
A25	-			
W26	3 (D, R, I)	$0.65^{+0.06}_{-0.05}$	$28.9^{+2.7}_{-2.4}$	$41.9^{+4.1}_{-3.7}$
A27	-			
M28	-			
V29	5 (2D, 2R, I)	$0.66^{+0.04}_{-0.04}$	$28.6^{+1.9}_{-1.7}$	$41.4^{+2.8}_{-2.6}$
V30	5 (2D, R, 2I)	$0.64^{+0.14}_{-0.03}$	$29.5^{+6.0}_{-1.3}$	$42.8^{+9.2}_{-2.0}$
V31	6 (2D, 3R, I)	$0.64^{+0.03}_{-0.03}$	$29.3^{+1.4}_{-1.4}$	$42.6^{+2.2}_{-2.1}$
I32	3 (D, 2R)	$0.68^{+0.03}_{-0.03}$	$27.7^{+1.0}_{-0.9}$	$40.0^{+2.2}_{-2.0}$
V33	1 (D)	$0.61^{+0.11}_{-0.09}$	$30.7^{+4.6}_{-4.1}$	$44.6^{+7.2}_{-6.4}$
G34	2 (R, I)	$0.72^{+0.03}_{-0.03}$	$25.8^{+1.7}_{-1.6}$	$37.1^{+2.5}_{-2.4}$
A35	4 (D, R, 2I)	$0.63^{+0.03}_{-0.03}$	$29.7^{+0.9}_{-0.9}$	$43.1^{+2.0}_{-1.9}$
T36	2 (I)	$0.69^{+0.07}_{-0.06}$	$27.2^{+3.2}_{-2.8}$	$39.4^{+4.9}_{-4.2}$
I37	3 (D, R, I)	$0.67^{+0.04}_{-0.04}$	$27.9^{+1.7}_{-1.6}$	$40.3^{+2.7}_{-2.5}$
G38	3 (D, R, I)	$0.69^{+0.03}_{-0.03}$	$27.0^{+1.6}_{-1.5}$	$39.0^{+2.4}_{-2.3}$
I39	3 (D, R, I)	$0.64^{+0.02}_{-0.02}$	$29.3^{+0.8}_{-0.7}$	$42.5^{+1.7}_{-1.6}$
K40	-			
L41	1 (R)	$0.65^{+0.04}_{-0.04}$	$28.9^{+1.9}_{-1.8}$	$41.8^{+3.0}_{-2.7}$
F42	1 (D, R)	$0.70^{+0.07}_{-0.13}$	$26.4^{+2.2}_{-4.1}$	$38.1^{+5.2}_{-9.5}$
K43	-			
K44	-			
F45	-			
T46	2 (R)	$0.65^{+0.05}_{-0.05}$	$29.0^{+2.4}_{-2.3}$	$42.1^{+3.7}_{-3.6}$
S47	-			
K48	-			
A49	3 (D, R, I)	$0.68^{+0.06}_{-0.05}$	$27.7^{+2.7}_{-2.2}$	$40.1^{+4.1}_{-3.3}$
S50	4 (D, 2R, I)	$0.66^{+0.03}_{-0.03}$	$28.5^{+1.4}_{-1.3}$	$41.2^{+2.1}_{-1.9}$

Table S6.2. Sidechain order parameters discussed in the main text. The rigid limits for the dipolar coupling constants are 1332Hz for G, 1005 Hz for K, 1317 Hz for Q, 1116 Hz for W.

#Residue	Amount of resolved crosspeaks	Bond	Order parameter	Amplitude (Motion on a cone) (°)	Amplitude (Motion in a cone) (°)
K8	2 (R, I)	Cε-Nε	$0.22^{+0.02}_{-0.02}$	$46.3^{+0.8}_{-0.9}$	$70.9^{+1.5}_{-1.8}$
Q15	1 (I)	Cδ-Nε2	$0.36^{+0.03}_{-0.03}$	$40.9^{+1.3}_{-1.3}$	$61.2^{+2.2}_{-2.2}$
W26	2 (D, R)	Cε2-Nε1	$0.75^{+0.10}_{-0.08}$	$24.1^{+5.0}_{-4.1}$	$34.7^{+7.4}_{-6.1}$
K40	4 (2D, R, I)	Cε-Nζ	$0.57^{+0.03}_{-0.03}$	$32.4^{+1.2}_{-1.2}$	$47.3^{+1.9}_{-1.9}$
K43	1 (R)	Cε-Nζ	$0.59^{+0.02}_{-0.04}$	$31.6^{+1.0}_{-0.9}$	$46.1^{+1.5}_{-1.4}$
K44	2 (R, I)	Cε-Nζ	$0.47^{+0.04}_{-0.04}$	$36.3^{+1.6}_{-1.7}$	$53.5^{+2.7}_{-2.7}$

The following plots include all the fits to the dephasing curves of all crosspeaks we analysed in alphabetical order. The left panels in the three columns show the best-fit REDOR curve with the experimental data points, and the right panels show χ^2_{red}. The crosspeak and the name of the mixing scheme are written in the title of the left panels, and the calculated effective dipolar interaction (and error) is written in the titles of the right panels. When ambiguous crosspeaks exist (not more than two options), both options are written in the title.

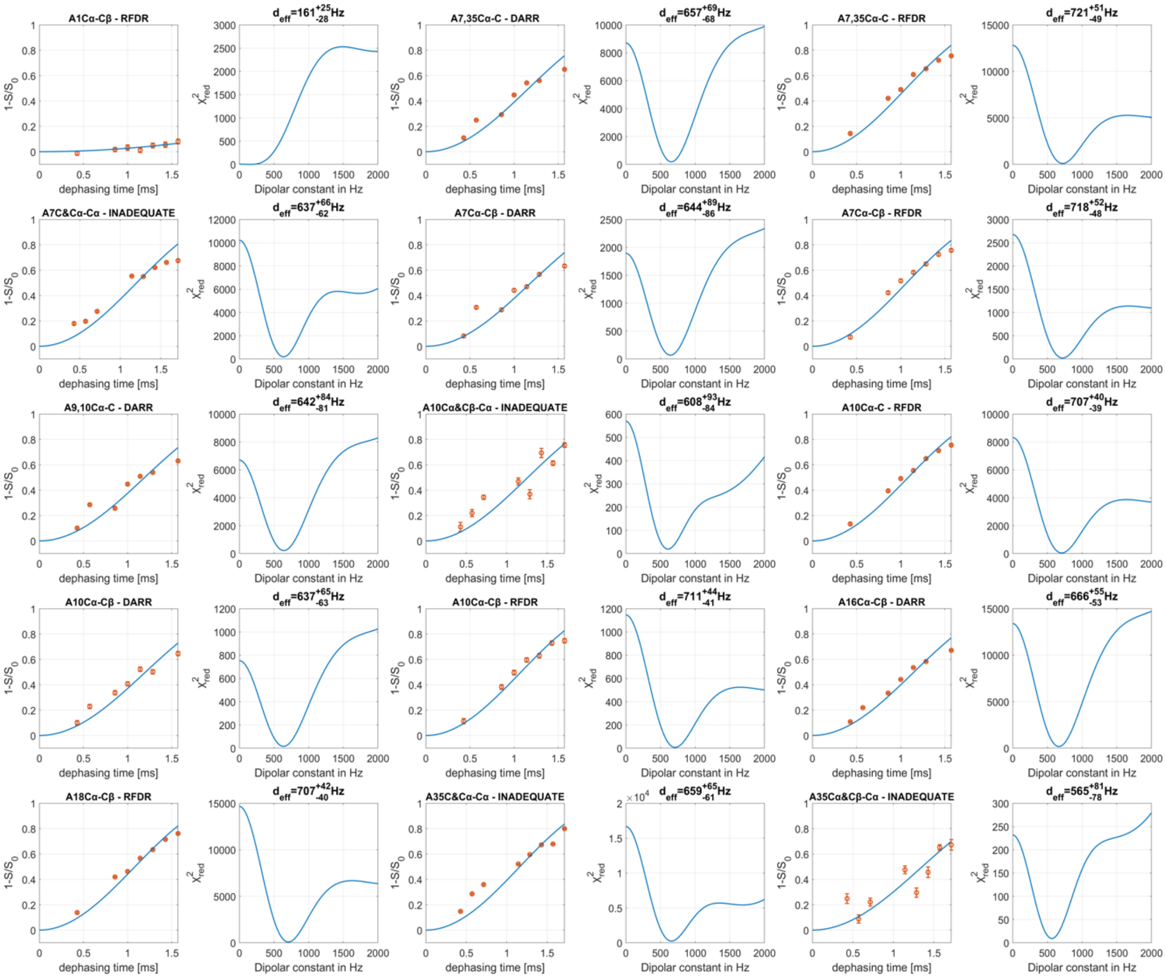

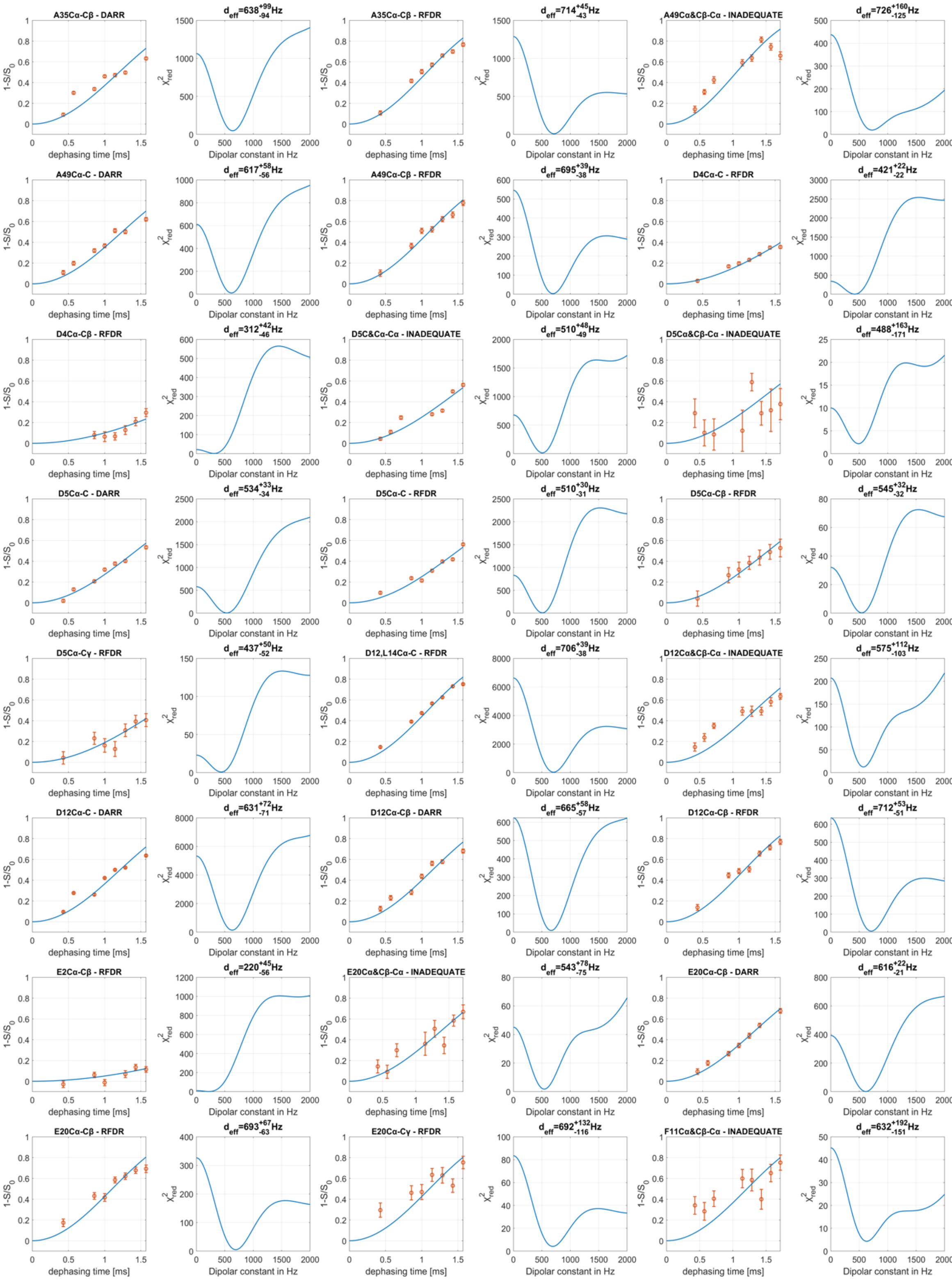

A35Cα-Cβ - DARR
$d_{eff}=638^{+99}_{-94}$Hz
A35Cα-Cβ - RFDR
$d_{eff}=714^{+45}_{-43}$Hz
A49Cα&Cβ-Cα - INADEQUATE
$d_{eff}=726^{+160}_{-125}$Hz
A49Cα-C - DARR
$d_{eff}=617^{+58}_{-56}$Hz
A49Cα-Cβ - RFDR
$d_{eff}=695^{+39}_{-38}$Hz
D4Cα-C - RFDR
$d_{eff}=421^{+22}_{-22}$Hz
D4Cα-Cβ - RFDR
$d_{eff}=312^{+42}_{-46}$Hz
D5C&Cα-Cα - INADEQUATE
$d_{eff}=510^{+48}_{-49}$Hz
D5Cα&Cβ-Cα - INADEQUATE
$d_{eff}=488^{+163}_{-171}$Hz
D5Cα-C - DARR
$d_{eff}=534^{+33}_{-34}$Hz
D5Cα-C - RFDR
$d_{eff}=510^{+30}_{-31}$Hz
D5Cα-Cβ - RFDR
$d_{eff}=545^{+32}_{-32}$Hz
D5Cα-Cγ - RFDR
$d_{eff}=437^{+50}_{-52}$Hz
D12,L14Cα-C - RFDR
$d_{eff}=706^{+39}_{-38}$Hz
D12Cα&Cβ-Cα - INADEQUATE
$d_{eff}=575^{+112}_{-103}$Hz
D12Cα-C - DARR
$d_{eff}=631^{+72}_{-71}$Hz
D12Cα-Cβ - DARR
$d_{eff}=665^{+58}_{-57}$Hz
D12Cα-Cβ - RFDR
$d_{eff}=712^{+53}_{-51}$Hz
E2Cα-Cβ - RFDR
$d_{eff}=220^{+45}_{-56}$Hz
E20Cα&Cβ-Cα - INADEQUATE
$d_{eff}=543^{+78}_{-75}$Hz
E20Cα-Cβ - DARR
$d_{eff}=616^{+22}_{-21}$Hz
E20Cα-Cβ - RFDR
$d_{eff}=693^{+67}_{-63}$Hz
E20Cα-Cγ - RFDR
$d_{eff}=692^{+132}_{-116}$Hz
F11Cα&Cβ-Cα - INADEQUATE
$d_{eff}=632^{+192}_{-151}$Hz
$1-S/S_0$
dephasing time [ms]
χ^2_{red}
Dipolar constant in Hz

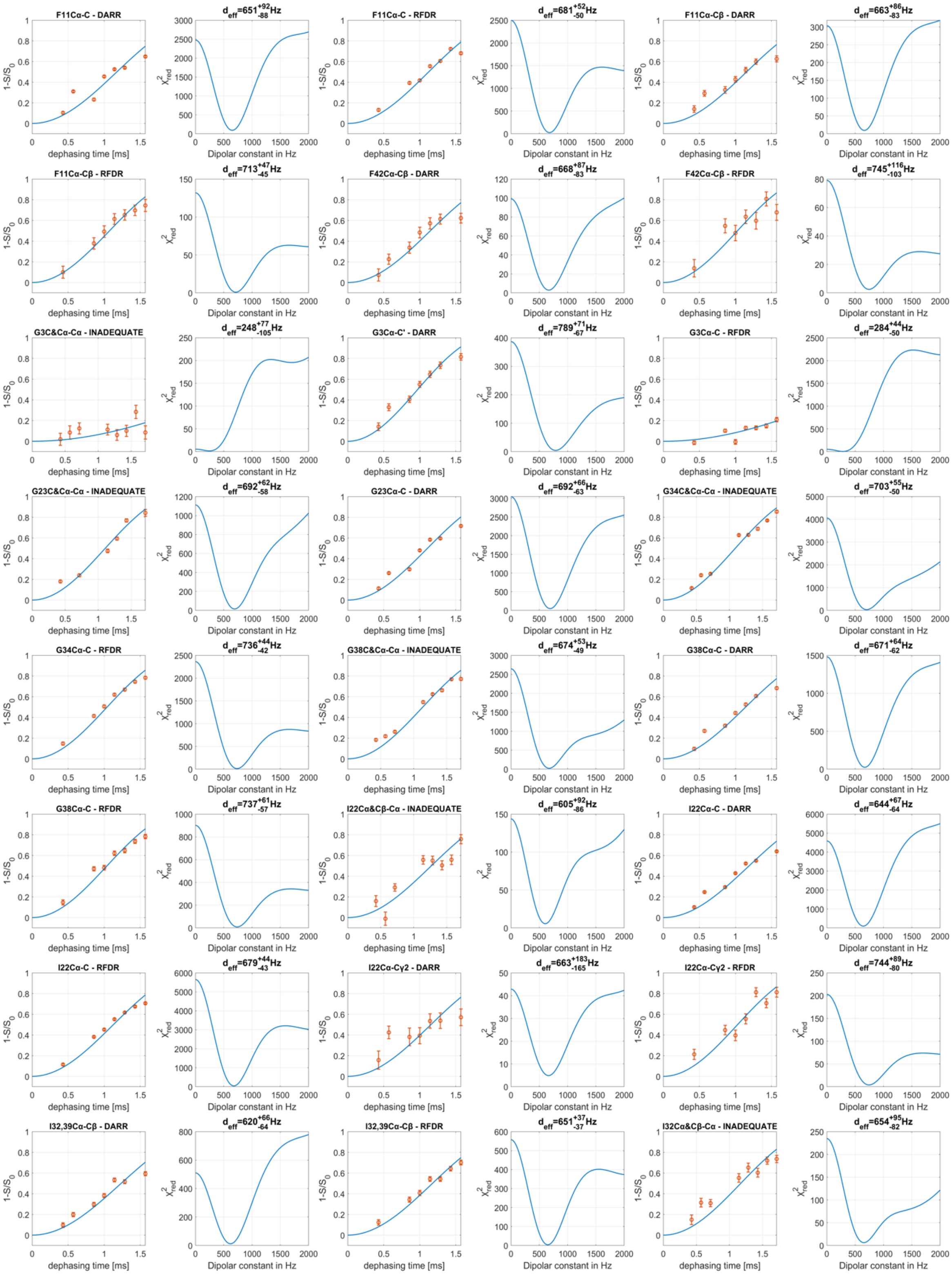
F11Cα-C - DARR
$d_{eff}=651^{+92}_{-88}$Hz
F11Cα-C - RFDR
$d_{eff}=681^{+52}_{-50}$Hz
F11Cα-Cβ - DARR
$d_{eff}=663^{+86}_{-83}$Hz
F11Cα-Cβ - RFDR
$d_{eff}=713^{+47}_{-45}$Hz
F42Cα-Cβ - DARR
$d_{eff}=668^{+87}_{-83}$Hz
F42Cα-Cβ - RFDR
$d_{eff}=745^{+116}_{-103}$Hz
G3C&Cα-Cα - INADEQUATE
$d_{eff}=248^{+77}_{-105}$Hz
G3Cα-C' - DARR
$d_{eff}=789^{+71}_{-67}$Hz
G3Cα-C - RFDR
$d_{eff}=284^{+44}_{-50}$Hz
G23C&Cα-Cα - INADEQUATE
$d_{eff}=692^{+62}_{-58}$Hz
G23Cα-C - DARR
$d_{eff}=692^{+66}_{-63}$Hz
G34C&Cα-Cα - INADEQUATE
$d_{eff}=703^{+55}_{-50}$Hz
G34Cα-C - RFDR
$d_{eff}=736^{+44}_{-42}$Hz
G38C&Cα-Cα - INADEQUATE
$d_{eff}=674^{+53}_{-49}$Hz
G38Cα-C - DARR
$d_{eff}=671^{+64}_{-62}$Hz
G38Cα-C - RFDR
$d_{eff}=737^{+61}_{-57}$Hz
I22Cα&Cβ-Cα - INADEQUATE
$d_{eff}=605^{+92}_{-86}$Hz
I22Cα-C - DARR
$d_{eff}=644^{+67}_{-64}$Hz
I22Cα-C - RFDR
$d_{eff}=679^{+44}_{-43}$Hz
I22Cα-Cγ2 - DARR
$d_{eff}=663^{+183}_{-165}$Hz
I22Cα-Cγ2 - RFDR
$d_{eff}=744^{+89}_{-80}$Hz
I32,39Cα-Cβ - DARR
$d_{eff}=620^{+66}_{-64}$Hz
I32,39Cα-Cβ - RFDR
$d_{eff}=651^{+37}_{-37}$Hz
I32Cα&Cβ-Cα - INADEQUATE
$d_{eff}=654^{+95}_{-82}$Hz
$1-S/S_0$
dephasing time [ms]
χ^2_{red}
Dipolar constant in Hz

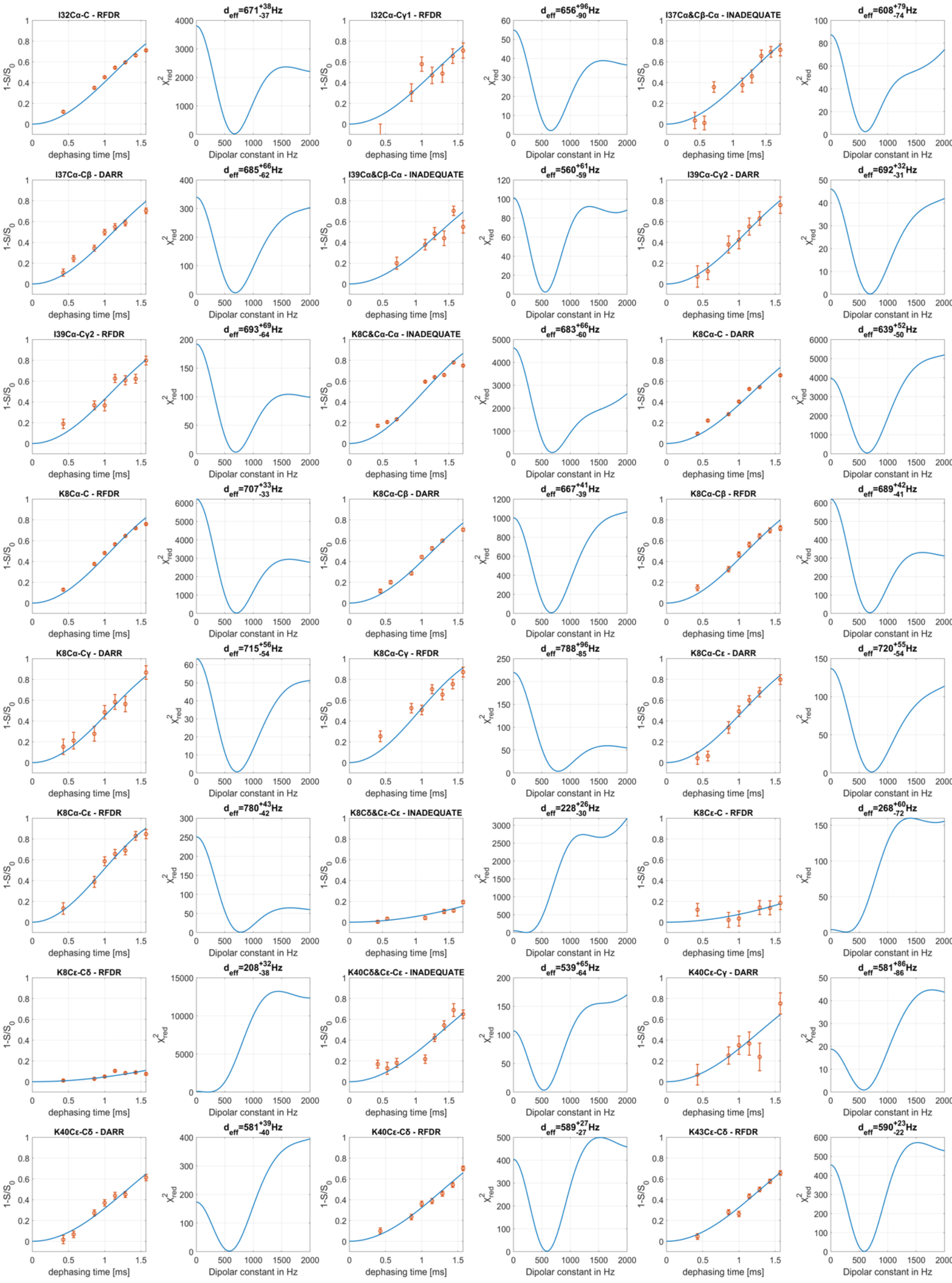
I32Cα-C - RFDR
d_eff=671+38-37Hz
I32Cα-Cγ1 - RFDR
d_eff=656+96-90Hz
I37Cα&Cβ-Cα - INADEQUATE
d_eff=608+79-74Hz
I37Cα-Cβ - DARR
d_eff=685+66-62Hz
I39Cα&Cβ-Cα - INADEQUATE
d_eff=560+61-59Hz
I39Cα-Cγ2 - DARR
d_eff=692+32-31Hz
I39Cα-Cγ2 - RFDR
d_eff=693+69-64Hz
K8C&Cα-Cα - INADEQUATE
d_eff=683+66-60Hz
K8Cα-C - DARR
d_eff=639+52-50Hz
K8Cα-C - RFDR
d_eff=707+33-33Hz
K8Cα-Cβ - DARR
d_eff=667+41-39Hz
K8Cα-Cβ - RFDR
d_eff=689+42-41Hz
K8Cα-Cγ - DARR
d_eff=715+56-54Hz
K8Cα-Cγ - RFDR
d_eff=788+96-85Hz
K8Cα-Cε - DARR
d_eff=720+55-54Hz
K8Cα-Cε - RFDR
d_eff=780+43-42Hz
K8Cδ&Cε-Cε - INADEQUATE
d_eff=228+26-30Hz
K8Cε-C - RFDR
d_eff=268+60-72Hz
K8Cε-Cδ - RFDR
d_eff=208+32-38Hz
K40Cδ&Cε-Cε - INADEQUATE
d_eff=539+65-64Hz
K40Cε-Cγ - DARR
d_eff=581+86-86Hz
K40Cε-Cδ - DARR
d_eff=581+39-40Hz
K40Cε-Cδ - RFDR
d_eff=589+27-27Hz
K43Cε-Cδ - RFDR
d_eff=590+23-22Hz
1-S/S0
dephasing time [ms]
χ2red
Dipolar constant in Hz

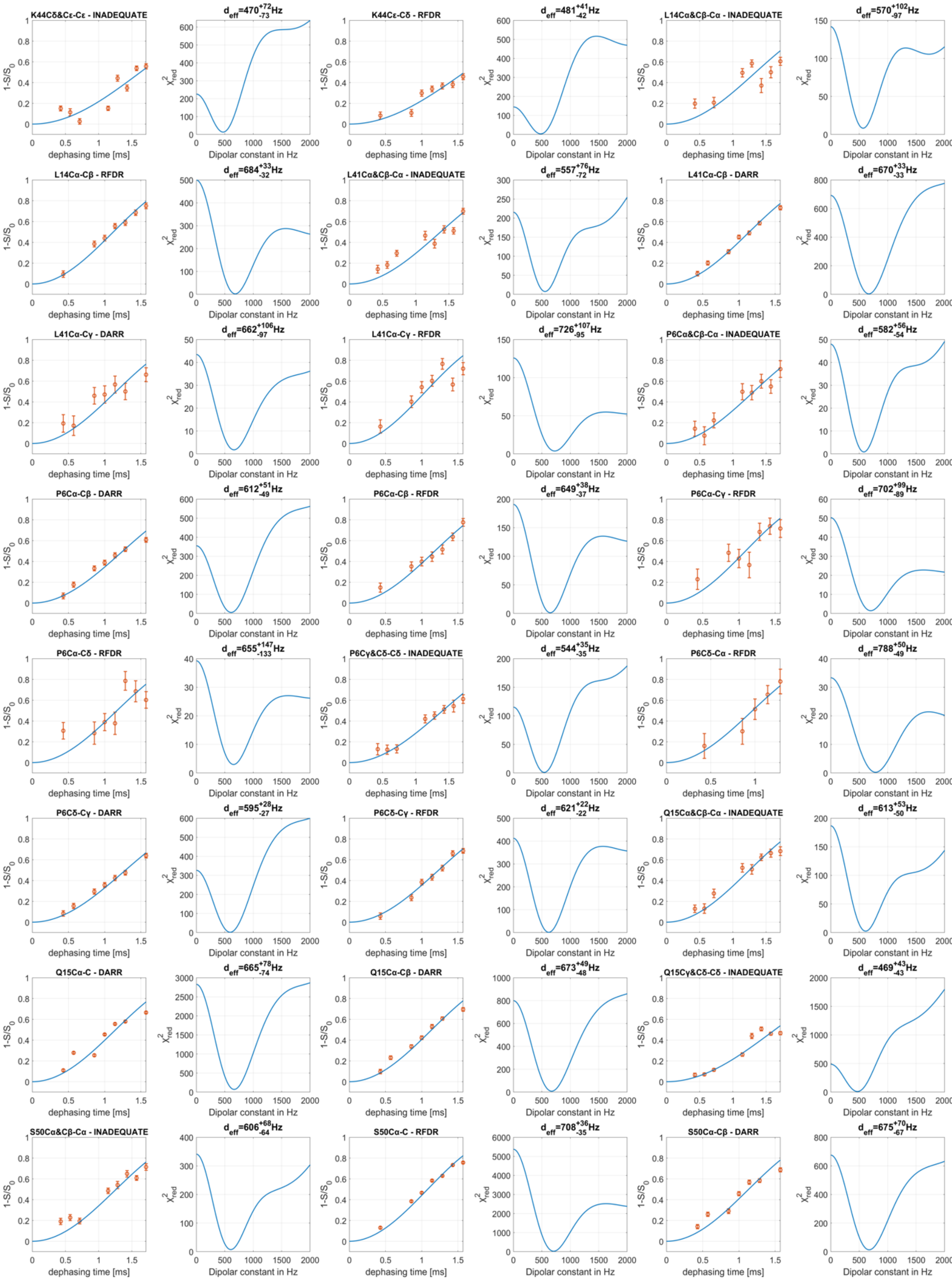
K44Cδ&Cε-Cε - INADEQUATE
$d_{eff}=470^{+72}_{-73}$Hz
K44Cε-Cδ - RFDR
$d_{eff}=481^{+41}_{-42}$Hz
L14Cα&Cβ-Cα - INADEQUATE
$d_{eff}=570^{+102}_{-97}$Hz
L14Cα-Cβ - RFDR
$d_{eff}=684^{+33}_{-32}$Hz
L41Cα&Cβ-Cα - INADEQUATE
$d_{eff}=557^{+76}_{-72}$Hz
L41Cα-Cβ - DARR
$d_{eff}=670^{+33}_{-33}$Hz
L41Cα-Cγ - DARR
$d_{eff}=662^{+106}_{-97}$Hz
L41Cα-Cγ - RFDR
$d_{eff}=726^{+107}_{-95}$Hz
P6Cα&Cβ-Cα - INADEQUATE
$d_{eff}=582^{+56}_{-54}$Hz
P6Cα-Cβ - DARR
$d_{eff}=612^{+51}_{-49}$Hz
P6Cα-Cβ - RFDR
$d_{eff}=649^{+38}_{-37}$Hz
P6Cα-Cγ - RFDR
$d_{eff}=702^{+99}_{-89}$Hz
P6Cα-Cδ - RFDR
$d_{eff}=655^{+147}_{-133}$Hz
P6Cγ&Cδ-Cδ - INADEQUATE
$d_{eff}=544^{+35}_{-35}$Hz
P6Cδ-Cα - RFDR
$d_{eff}=788^{+50}_{-49}$Hz
P6Cδ-Cγ - DARR
$d_{eff}=595^{+28}_{-27}$Hz
P6Cδ-Cγ - RFDR
$d_{eff}=621^{+22}_{-22}$Hz
Q15Cα&Cβ-Cα - INADEQUATE
$d_{eff}=613^{+53}_{-50}$Hz
Q15Cα-C - DARR
$d_{eff}=665^{+78}_{-74}$Hz
Q15Cα-Cβ - DARR
$d_{eff}=673^{+49}_{-48}$Hz
Q15Cγ&Cδ-Cδ - INADEQUATE
$d_{eff}=469^{+43}_{-43}$Hz
S50Cα&Cβ-Cα - INADEQUATE
$d_{eff}=606^{+68}_{-64}$Hz
S50Cα-C - RFDR
$d_{eff}=708^{+36}_{-35}$Hz
S50Cα-Cβ - DARR
$d_{eff}=675^{+70}_{-67}$Hz
1-S/S0
dephasing time [ms]
χ^2_{red}
Dipolar constant in Hz

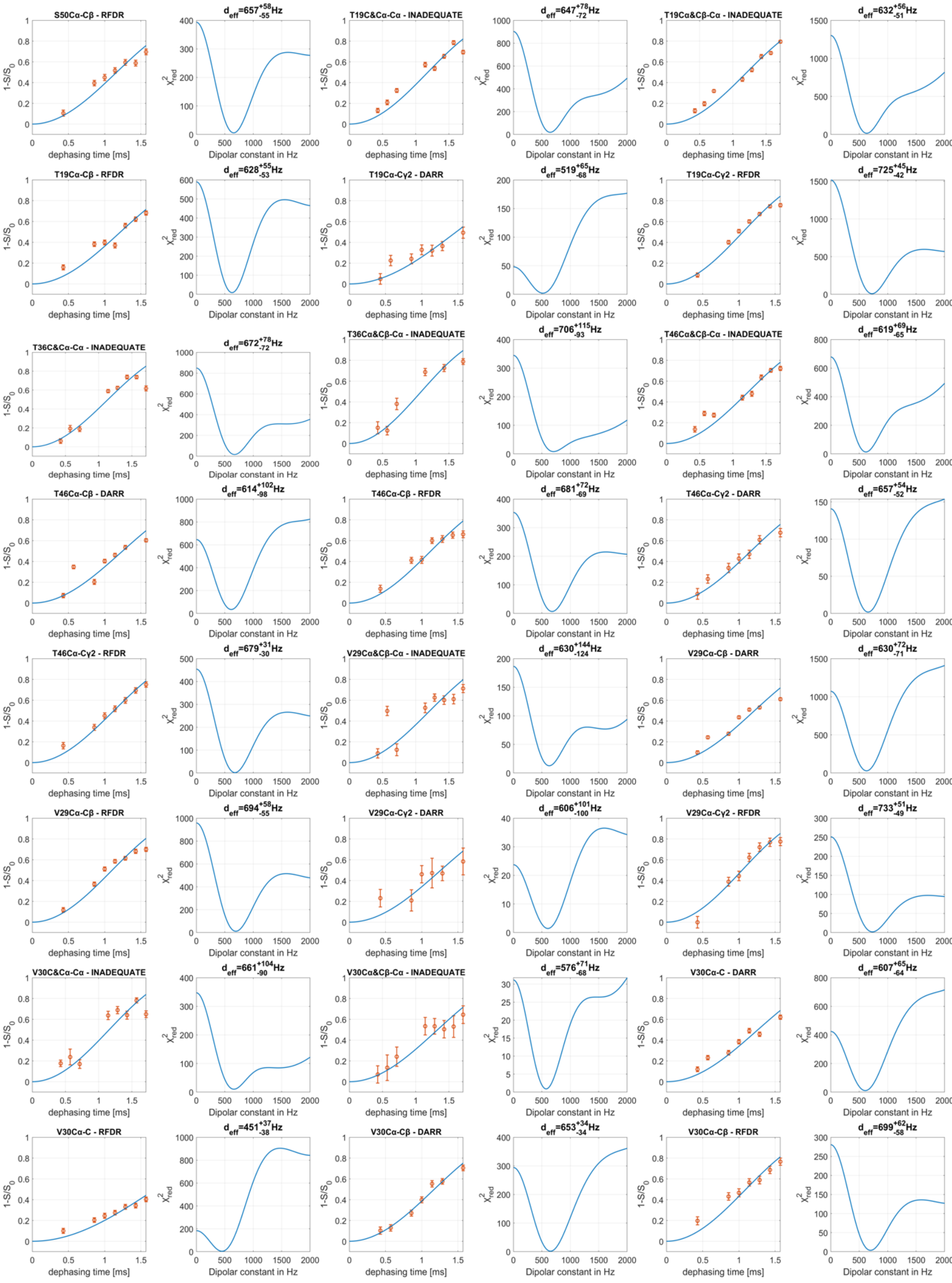
S50Cα-Cβ - RFDR
$d_{eff}=657^{+58}_{-55}$Hz
T19C&Cα-Cα - INADEQUATE
$d_{eff}=647^{+78}_{-72}$Hz
T19Cα&Cβ-Cα - INADEQUATE
$d_{eff}=632^{+56}_{-51}$Hz
T19Cα-Cβ - RFDR
$d_{eff}=628^{+55}_{-53}$Hz
T19Cα-Cγ2 - DARR
$d_{eff}=519^{+65}_{-68}$Hz
T19Cα-Cγ2 - RFDR
$d_{eff}=725^{+45}_{-42}$Hz
T36C&Cα-Cα - INADEQUATE
$d_{eff}=672^{+78}_{-72}$Hz
T36Cα&Cβ-Cα - INADEQUATE
$d_{eff}=706^{+115}_{-93}$Hz
T46Cα&Cβ-Cα - INADEQUATE
$d_{eff}=619^{+69}_{-65}$Hz
T46Cα-Cβ - DARR
$d_{eff}=614^{+102}_{-98}$Hz
T46Cα-Cβ - RFDR
$d_{eff}=681^{+72}_{-69}$Hz
T46Cα-Cγ2 - DARR
$d_{eff}=657^{+54}_{-52}$Hz
T46Cα-Cγ2 - RFDR
$d_{eff}=679^{+31}_{-30}$Hz
V29Cα&Cβ-Cα - INADEQUATE
$d_{eff}=630^{+144}_{-124}$Hz
V29Cα-Cβ - DARR
$d_{eff}=630^{+72}_{-71}$Hz
V29Cα-Cβ - RFDR
$d_{eff}=694^{+58}_{-55}$Hz
V29Cα-Cγ2 - DARR
$d_{eff}=606^{+101}_{-100}$Hz
V29Cα-Cγ2 - RFDR
$d_{eff}=733^{+51}_{-49}$Hz
V30C&Cα-Cα - INADEQUATE
$d_{eff}=661^{+104}_{-90}$Hz
V30Cα&Cβ-Cα - INADEQUATE
$d_{eff}=576^{+71}_{-68}$Hz
V30Cα-C - DARR
$d_{eff}=607^{+65}_{-64}$Hz
V30Cα-C - RFDR
$d_{eff}=451^{+37}_{-38}$Hz
V30Cα-Cβ - DARR
$d_{eff}=653^{+34}_{-34}$Hz
V30Cα-Cβ - RFDR
$d_{eff}=699^{+62}_{-58}$Hz
$1-S/S_0$
dephasing time [ms]
χ^2_{red}
Dipolar constant in Hz

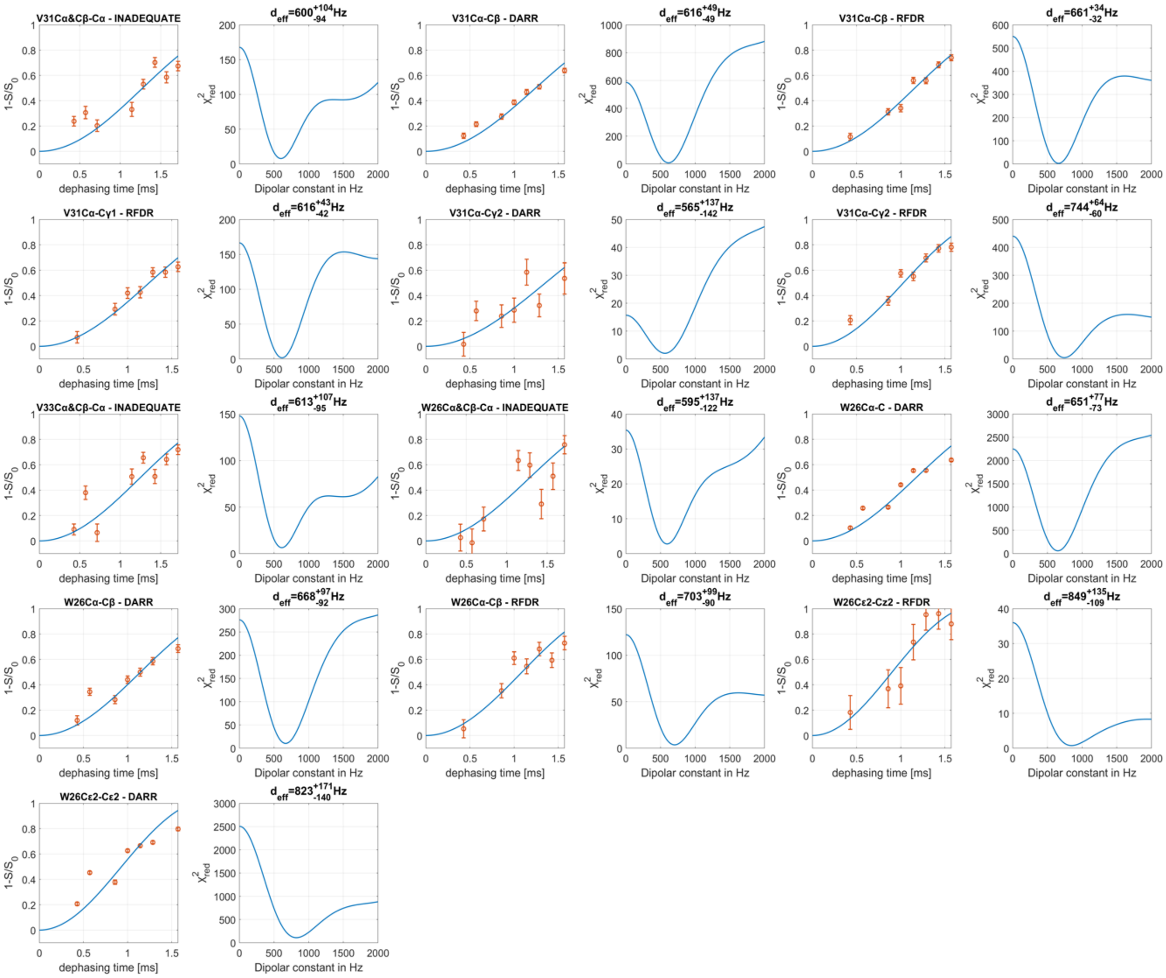

S7 – Filtered INADEQUATE

The application of the REDOR block causes the dephasing of signals originating from carbons in close proximity to nitrogen. At the longest dephasing time that was used in our experiments, 1.714ms, approximately 80% of their signal decayed, effectively eliminating it from the spectrum.

Figure S7 shows a comparison between the same 'S' INADEQUATE-REDOR crosspeaks of threonine residues (19, 36, 46) in the fd-Y21M phage coat protein through different dephasing times. The figure shows how the signals of double-quantum Cα+Cβ crosspeaks with Cα in the acquisition dimension decay with increased dephasing times, while the crosspeaks with Cβ in the acquisition SQ dimension are not affected by the pulses, only by relaxation. The resulting edited spectrum has decreased spectral congestion while still containing information about the Cα chemical shifts encoded in the DQ dimension.

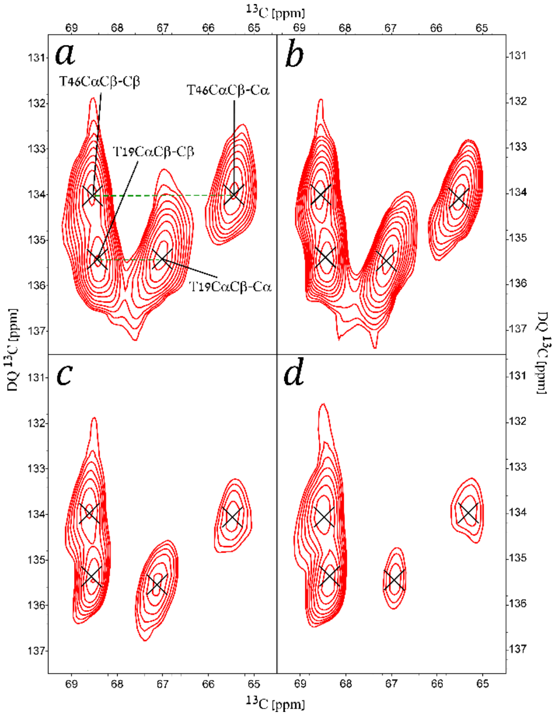

Figure S7. Extractions of INADEQUATE-REDOR 'S' spectra showing threonine DQ correlations with (a)-(d) corresponding to dephasing times of 429μs, 571μs, 1571μs and 1714μs, respectively. Contour levels are drawn at multiples of 1.3 with the lowest matching an SNR of 8.

In order to assess the effects of relaxation due to the addition of the REDOR block, we compared the SNR of 15 randomly selected non-ambiguous crosspeaks in both S and S_0 spectra. The SNR values are detailed in the following table.

	SNR		
Crosspeak	S_0 (0.429ms)	S (1.714ms)	Signal reduction
E2Cα&Cβ- Cβ	25	16	36%
P6Cγ&Cδ- Cγ	17	15	12%
K8Cγ&Cδ- Cδ	46	53	-15%
K8Cγ&Cδ- Cγ	33	29	12%
D12Cα&Cβ- Cβ	21	22	-5%
I37Cγ1&Cδ1- Cδ1	56	31	45%
Q15Cα&Cβ- Cβ	28	25	11%
Q15Cγ&Cδ- Cγ	33	32	3%
I22Cγ1&Cδ1- Cδ1	41	37	10%
I37Cβ&Cγ2- Cβ	31	15	52%
I37Cγ1&Cδ1- Cγ1	24	23	4%
I37Cβ&Cγ2- Cγ2	67	43	36%
I37Cγ1&Cδ1- Cδ1	56	31	45%
I39Cγ1&Cδ1- Cγ1	14	17	-21%
I39Cγ1&Cδ1- Cδ1	75	71	5%
T46Cα&Cβ- Cβ	52	59	-13%

On average, the change in the SNR is a decrease of 13%.

SI bibliography

(1) Lee, W.; Tonelli, M.; Markley, J. L. NMRFAM-SPARKY: Enhanced Software for Biomolecular NMR Spectroscopy. *Bioinformatics* **2015**, *31* (8), 1325–1327. https://doi.org/10.1093/bioinformatics/btu830.

(2) Lee, W.; Rahimi, M.; Lee, Y.; Chiu, A. POKY: A Software Suite for Multidimensional NMR and 3D Structure Calculation of Biomolecules. *Bioinformatics* **2021**, *37* (18), 3041–3042. https://doi.org/10.1093/bioinformatics/btab180.

(3) Li, Y.; Evans, J. N. S. The Importance of XY-8 Phase Cycling in the Rotational-Echo Double-Resonance Experiment with Total Sideband Suppression. *J. Magn. Reson. A* **1995**, *116* (2), 150–155. https://doi.org/10.1006/jmra.1995.0003.

(4) Aharoni, T.; Goldbourt, A. Rapid Automated Determination of Chemical Shift Anisotropy Values in the Carbonyl and Carboxyl Groups of Fd-Y21m Bacteriophage Using Solid State NMR. *J. Biomol. NMR* **2018**, *72* (1), 55–67. https://doi.org/10.1007/s10858-018-0206-1.

(5) Koehl, P. Linear Prediction Spectral Analysis of NMR Data. *Prog. Nucl. Magn. Reson. Spectrosc.* **1999**, *34* (3), 257–299. https://doi.org/10.1016/S0079-6565(99)00002-3.

(6) Bak, M.; Rasmussen, J. T.; Nielsen, N. C. SIMPSON: A General Simulation Program for Solid-State NMR Spectroscopy. *J. Magn. Reson.* **2000**, *147* (2), 296–330. https://doi.org/10.1006/jmre.2000.2179.
